# Artificial intelligence in healthcare and medicine: clinical applications, therapeutic advances, and future perspectives

**DOI:** 10.1186/s40001-025-03196-w

**Published:** 2025-09-23

**Authors:** Yosri A. Fahim, Ibrahim W. Hasani, Samer Kabba, Waleed Mahmoud Ragab

**Affiliations:** 1 Department of Basic Medical Sciences, Health Sector, Galala University, Suez, 43511 Egypt; 2 Faculty of Pharmacy, Al-Andalus University for Medical Sciences, Qadmus, Tartus, Syrian Arab Republic; 3 Department of Anatomy and Embryology, Faculty of Medicine, Galala University, Suez, 43511 Egypt

**Keywords:** Artificial intelligence, Healthcare systems, Personalized medicine, Diagnostics, Ethical challenges

## Abstract

Healthcare systems worldwide face growing challenges, including rising costs, workforce shortages, and disparities in access and quality, particularly in low- and middle-income countries. Artificial intelligence (AI) has emerged as a transformative tool capable of addressing these issues by enhancing diagnostics, treatment planning, patient monitoring, and healthcare efficiency. AI’s role in modern medicine spans disease detection, personalized care, drug discovery, predictive analytics, telemedicine, and wearable health technologies. Leveraging machine learning and deep learning, AI can analyze complex data sets, including electronic health records, medical imaging, and genomic profiles, to identify patterns, predict disease progression, and recommend optimized treatment strategies. AI also has the potential to promote equity by enabling cost-effective, resource-efficient solutions in low-resource and remote settings, such as mobile diagnostics, wearable biosensors, and lightweight algorithms. Successful deployment requires addressing critical challenges, including data privacy, algorithmic bias, model interpretability, regulatory oversight, and maintaining human clinical oversight. Emphasizing scalable, ethical, and evidence-driven implementation, key strategies include clinician training in AI literacy, adoption of resource efficient tools, global collaboration, and robust regulatory frameworks to ensure transparency, safety, and accountability. By complementing rather than replacing healthcare professionals, AI can reduce errors, optimize resources, improve patient outcomes, and expand access to quality care. This review emphasizes the responsible integration of AI as a powerful catalyst for innovation, sustainability, and equity in healthcare delivery worldwide.

## Introduction

The healthcare industry is undergoing a profound transformation driven by escalating costs, workforce shortages, and increasing demands from aging populations. Globally, healthcare systems face critical challenges, such as limited access to care, inefficiencies, and high expenses [1]. These issues are particularly severe in low- and middle-income countries, where shortages of trained healthcare professionals, inadequate infrastructure, and limited diagnostic capabilities often result in delayed disease detection, suboptimal treatments, and poorer patient outcomes. Addressing these challenges requires innovative, scalable solutions capable of improving efficiency, expanding access, and enhancing quality of care across diverse healthcare environments [2].

Artificial intelligence (AI) is the study of how computers can learn to solve problems using symbolic language [3]. Many fields, including medicine, pharmaceutics, and more, have benefited from its development, and it has become a core research method for resolving issues [4]. It offers promising tools for transforming healthcare delivery through advanced data analysis and decision support. AI systems, powered by machine learning (ML), can process vast amounts of patient information, including medical histories, test results, treatment responses, and clinical guidelines, to develop personalized care strategies [5]. These technologies assist clinicians by recommending optimal therapies based on individual health profiles and by continuously monitoring vital signs to detect early signs of complications. A key advantage of AI is its ability to identify hidden patterns in large data sets, enabling predictions about disease progression, treatment outcomes, and patient risk factors [6]. Such predictive capabilities facilitate early interventions, preventive care, and more precise allocation of resources. Despite its potential, the adoption of AI in healthcare faces significant challenges, including ethical considerations regarding patient privacy, data security, and mitigating algorithmic biases arising from historical data [7].

Ensuring healthcare providers receive adequate training is critical to maximizing AI benefits, supporting rather than replacing clinical judgment [8]. Traditional diagnostic methods depend heavily on human expertise, are susceptible to fatigue, and are subject to subjective interpretation. AI enhances diagnostic accuracy and speeds decision-making by integrating diverse data sources, such as electronic health records, medical imaging, genomic profiles, and scientific literature [9]. Deep learning (DL) algorithms, for example, have shown remarkable success in detecting abnormalities across various imaging modalities, including X-rays, CT scans, MRIs, and pathology slides [10]. While many reviews have explored AI’s applications in diagnostics, treatment planning, and clinical decision support, fewer have addressed AI’s potential to reduce healthcare disparities. Limited research focuses on adapting AI technologies to improve access and quality of care in underserved and resource-constrained settings, where geographic barriers and workforce shortages exacerbate health inequities.

This review comprehensively analyzes AI's contributions to healthcare, emphasizing advancements in deep learning, generative modeling, predictive analytics, and system integration. Moreover, it highlights AI's role in promoting healthcare equity through adaptable, cost-effective solutions such as telemedicine, mobile diagnostics, wearable biosensors, and low-computation algorithms suitable for low-resource environments. By examining high-resource and resource-limited contexts, this work aims to inform future research, policy decisions, and strategic implementations that harness AI to create more equitable, accessible, and adequate healthcare worldwide.

## Need for the study

AI is rapidly transforming healthcare by enabling advances in diagnostics, personalized medicine, treatment planning, and operational efficiency. Despite growing interest and numerous studies, existing reviews often focus narrowly on specific applications or technologies. In addition, there is limited examination of AI’s potential to address disparities in healthcare access and quality across diverse resource settings. This comprehensive review aims to fill these gaps by analyzing the breadth of AI applications in medicine while emphasizing equitable healthcare delivery. By synthesizing recent advances and challenges, this study provides critical insights to guide future research, clinical implementation, and policymaking.

## Historical overview of artificial intelligence in healthcare

**Fig. 1:**
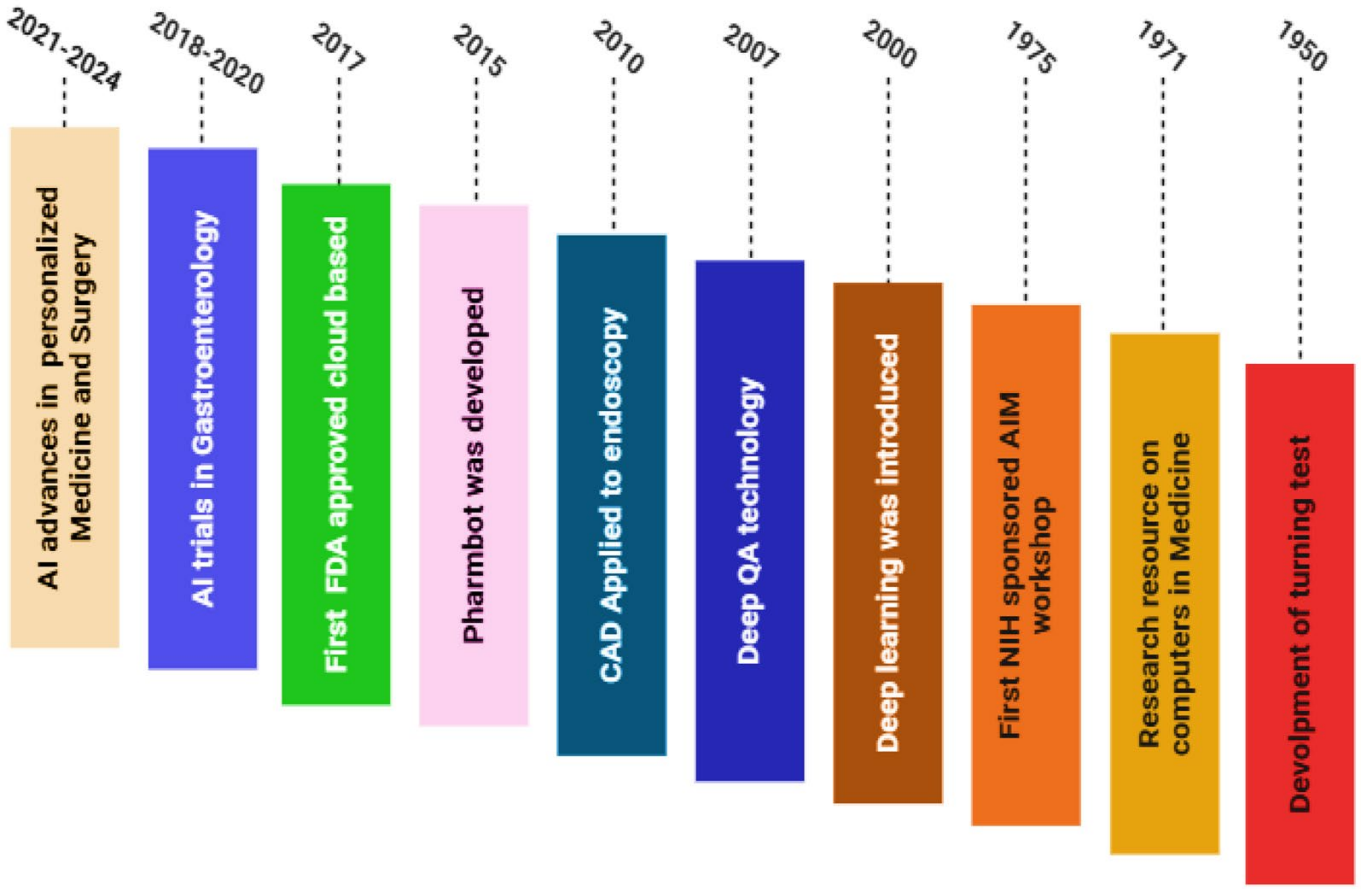
History of artificial intelligence in healthcare

The integration of AI into Healthcare has evolved significantly since the mid-twentieth century. In 1950, AI made its first notable contribution to medicine during research on shifting tests. The impact of computational intelligence was further highlighted in 1975 with the development of an early prototype study on computer applications in medicine. Since then, AI’s reach has expanded, particularly with the advent of DeepQA software in 2007, which marked a notable advancement in AI-driven analysis. Early applications such as computer-aided detection (CAD) in endoscopy appeared in 2010, followed By the development of Pharmbot software in 2015. A landmark event was the 2017 launch of a cloud-based deep learning application that received FDA approval, signifying regulatory acceptance of AI tools in clinical practice. Between 2018 and 2020, numerous AI trials in gastroenterology showcased the growing adoption of AI, while a dramatic transformation in pharmaceutical supply chain management further demonstrated AI’s industrial impact. The period from 2021 to 2024 saw accelerated AI diagnostics deployment during the COVID-19 pandemic, advancements in personalized medicine, development of explainable AI for clinical transparency, and integration of AI with robotic surgery and telemedicine [11]. Figure 1 illustrates the timeline of key milestones and innovations marking the evolution of AI in healthcare, contextualizing its growing role in modern medicine.

## Methodology

The methodology of this review involved a comprehensive and systematic search of scientific literature to gather current evidence on the diagnostic, therapeutic, and equity-oriented applications of artificial intelligence in healthcare. A targeted search was carried out across major databases, including PubMed, Scopus, Web of Science, IEEE Xplore, and Google Scholar. Keywords used in various combinations included “artificial intelligence,” “machine learning,” “deep learning,” “natural language processing,” “healthcare,” “low-resource settings,” and “health equity.” Only English-language articles published Between 2015 and 2025 were considered. Studies were selected based on their relevance to AI applications in healthcare, with a preference for original research, clinical trials, implementation studies, and high-impact reviews that reported measurable clinical or operational outcomes. Exclusion criteria included studies outside the healthcare domain, articles lacking a clear AI component, editorials, opinion pieces, and publications without sufficient methodological or application detail. Following an initial screening of titles and abstracts, eligible articles underwent full-text review to confirm relevance and quality. Data were extracted regarding the AI techniques employed, the healthcare domain addressed, target populations, implementation settings, and reported outcomes. Special emphasis was placed on identifying AI applications that demonstrated adaptability to low-resource contexts or contributed to reducing disparities in healthcare access. The included studies were organized into thematic categories covering diagnostics, treatment planning, oncology, drug discovery, rehabilitation, and digital health innovations. Within each category, applications were further analyzed for innovation, scalability, translational potential, and scientific quality.

## Foundational AI technologies

### Machine learning

ML is a crucial subset of artificial intelligence that enables computers to recognize patterns and acquire knowledge from data without direct programming [12]. It is extensively utilized in healthcare for disease classification, patient risk stratification, and outcome prediction [13]. It includes various learning paradigms such as supervised learning, wherein models are trained on data sets with known outcomes to forecast future instances; unsupervised learning, which identifies concealed patterns in unlabeled health data to uncover new disease subtypes or patient cohorts; and reinforcement learning, which determines optimal treatment strategies through trial and error, though it is less frequently employed in clinical environments [14]. By facilitating the examination of extensive and intricate healthcare data sets, ML enhances the accuracy and personalization of medical decision-making.

### Deep learning

DL, a specialized subset of ML, employs multilayered artificial neural networks to represent complex and high-dimensional healthcare data. This methodology has revolutionized medical AI applications owing to its exceptional capacity to manage complexity, especially in image and sequence data analysis [15]. Convolutional Neural Networks (CNNs) are widely used in medical imaging to detect and segment anomalies in X-rays, CT scans, MRIs, and pathology slides with exceptional precision [16]. Recurrent Neural Networks (RNNs) and transformer topologies are proficient in processing sequential data, including electronic health records and physiological time-series signals, improving patient monitoring and result prediction [17]. Ongoing enhancements in these designs enhance feature extraction and predictive efficacy, enabling more precise and automated clinical insights.

### Natural language processing

Natural Language Processing (NLP) enables AI systems to comprehend, evaluate, and produce human language, extracting valuable insights from unstructured clinical documents, such as physicians'notes, discharge summaries, radiology reports, and scientific publications [18]. NLP in healthcare automates the extraction of clinical ideas, improves the identification of adverse events, and facilitates patient communication via chatbots and virtual assistants [19]. Advancements in transformer-based NLP models, such as BERT and GPT, have markedly enhanced the contextual comprehension of medical language, facilitating advanced applications like clinical trial matching and a thorough summary of medical papers. These enhancements enable efficient data processing and improved clinical decision-making [20].

### Generative models

Generative models, such as generative adversarial networks (GANs) and variational autoencoders, have offered innovative functionalities in healthcare AI by producing realistic synthetic data that emulates genuine patient information [21]. These models are essential for enhancing restricted data sets, especially in medical imaging, thereby increasing the resilience and generalizability of AI models [22]. In addition to imaging, generative models aid drug discovery by creating innovative molecular structures and simulating patient illness trajectories to predict progression patterns [23]. Although these advancements greatly expedite the advancement of healthcare AI, meticulous consideration is necessary to address issues associated with data quality, privacy, and potential biases present in synthetic data sets.

## AI applications in medicine and healthcare

### Integration of AI in electronic health records

The integration of AI into electronic health records (EHRs) represents a pivotal advancement in the management and utilization of patient data for clinical decision-making [24]. By analyzing electronic health records and real time data, AI systems detect diseases earlier and more accurately than traditional approaches. EHRs, which encompass comprehensive patient histories, laboratory results, and treatment documentation, generate extensive data sets that AI systems can analyze to extract actionable insights [25]. AI-powered EHR systems can identify patterns related to disease onset, treatment effectiveness, and patient safety issues more quickly than traditional methods. For example, algorithms can monitor medication interactions and alert providers to potential adverse events in real time. In addition, AI applications in EHR can improve disease surveillance, support population health management, and optimize resource allocation by predicting hospital admissions or staff workload requirements [26]. ML models, such as those investigated by *Rajkomar *et al*.,* have Been applied to EHRs to predict critical clinical outcomes including in-hospital mortality, hospital readmissions, and the onset of sepsis with higher accuracy than conventional scoring systems, Their study notably demonstrated that DL techniques could process unstructured EHR data, such as free-text clinical notes, achieving predictive accuracies exceeding 85%, thereby significantly outperforming manual methods [27]. *Friedman *et al*.,* illustrated how NLP can extract clinically relevant information such as symptom descriptions or medication adjustments from physicians’ notes, thereby supporting real-time clinical decision-making. This capacity is particularly valuable for risk stratification, where AI identifies high-risk individuals who may benefit from preventive care interventions [28]. *Obermeyer *et al*.,* demonstrated the utility of predictive analytics in population health management by identifying patients requiring proactive care strategies [29]. In addition to clinical applications, AI-driven EHR systems contribute to operational efficiency by automating administrative processes, such as medical coding and billing. This reduces the documentation burden on healthcare providers, as emphasized by *Davenport and Kalakota* [30]. Nonetheless, several challenges hinder the seamless integration of AI into EHR systems. These include issues of data interoperability across disparate platforms and concerns regarding data privacy and security, as highlighted by *Abouelmehdi *et al*.* [31].

### Personalized medicine and treatment plans

**Fig. 2:**
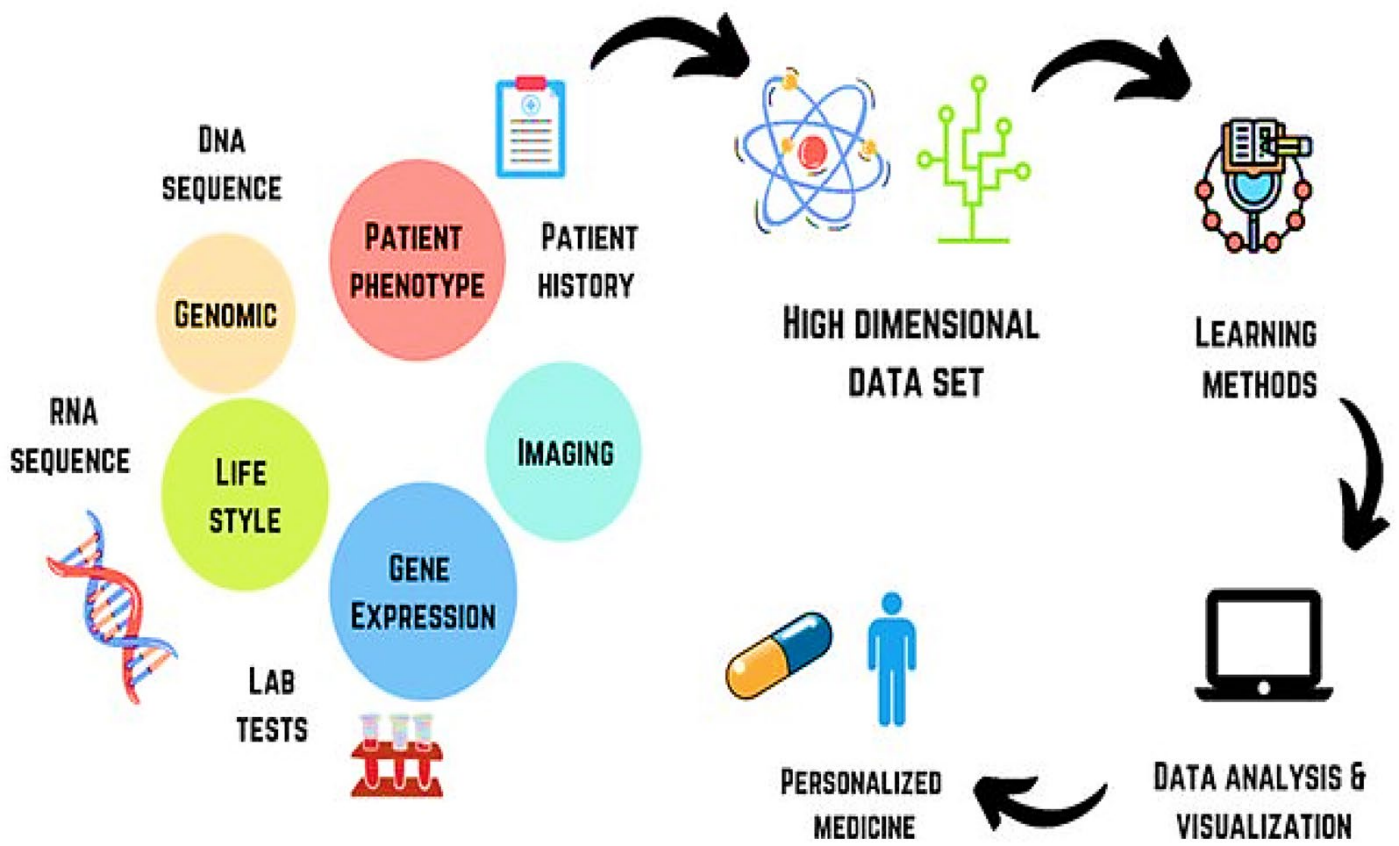
AI in acquiring and analyzing data of a patient in personalizing the treatment [34]

AI's capacity to design personalized medicine and treatment regimens signifies a critical paradigm shift from standardized clinical protocols to individualized patient care [32]. Through the integration and analysis of patient-specific data, such as genomic sequences, lifestyle patterns, and detailed medical histories, AI systems are capable of generating customized therapeutic strategies [33], as shown in Fig. 2 [34]. Moreover, AI enables personalized medicine by tailoring treatments to individual profiles. *Collins *et al*.,* demonstrated that AI algorithms can identify genetic mutations associated with rare diseases and subsequently recommend targeted therapies, enhancing patient response rates By up to 30% when compared to conventional treatment approaches [35]. In contrast to broad spectrum interventions, these AI-driven plans are designed to minimize adverse effects while maximizing therapeutic efficacy, especially in chronic conditions, such as diabetes and hypertension. The clinical utility of AI extends further, as evidenced by *Patel *et al*.,* who illustrated the application of AI in the real-time adjustment of insulin dosages for diabetic patients as by utilizing data from continuous glucose monitoring systems, AI facilitates more precise glycemic control, thereby highlighting its transformative potential in tailoring interventions to individual biological profiles [36]. In addition to therapeutic personalization, AI enhances treatment optimization by integrating imaging modalities with patient-specific data. *Gupta *et al*.,* demonstrated how AI can synthesize MRI data with genetic markers to develop individualized radiotherapy schedules for Brain tumor patients, resulting in a 25% increase in tumor control rates [37]. This integrative approach allows for treatments that are not only personalized but also dynamically responsive to evolving patient conditions, offering a level of clinical adaptability that surpasses traditional, static treatment protocols. Beyond tailoring treatment, AI’s predictive capacity enables early identification of risks, laying the groundwork for proactive disease prevention.

### Predictive analytics for disease prevention

Predictive analytics powered by AI offers a proactive approach to disease prevention by identifying health risks before clinical symptoms emerge [38]. Through the processing of population health data, environmental variables, and behavioral trends, AI models are capable of forecasting both disease outbreaks and individual susceptibility. A study by *Lee *et al*.* demonstrated that AI could predict influenza epidemics with 85% accuracy by analyzing social media activity and weather patterns, thereby enabling the timely implementation of vaccination campaigns [39]. In addition, AI can evaluate cardiovascular risk using data from routine blood tests and lifestyle surveys, providing clinicians with alerts that support early interventions, such as prescribing statins or recommending dietary modifications [40]. This anticipatory capacity represents a significant shift in healthcare, transitioning the model from reactive treatment to pre-emptive care, with the potential to reduce both morbidity and overall healthcare expenditures. Such predictive tools complement diagnostic imaging, where AI further enhances precision in detecting and characterizing diseases.

### AI in diagnostics and medical imaging

AI significantly enhances medical imaging by detecting abnormalities with exceptional sensitivity [41]. Unlike conventional diagnostic methods that rely heavily on human interpretation, AI systems are capable of analyzing X-rays, CT scans, and MRIs to accurately identify conditions, such as fractures and tumors [42]. The integration of AI in the analysis of chest radiographs for Lung cancer screening has become increasingly important, particularly given the global prevalence of lung cancer and the Limitations associated with traditional screening methods. Evidence indicates that AI algorithms generally achieve higher sensitivity ranging from 56.4% to 95.7% compared to radiologists, whose sensitivity ranges from 23.2% to 76% while maintaining comparable specificity [43]. This enhanced diagnostic precision enables earlier and more accurate detection, thereby facilitating timely treatment and improving patient outcomes, particularly in critical conditions, such as stroke and cancer. AI tools have surpassed radiologists in detecting cancers from imaging, as reviewed by *Litjens *et al*.* [44]. DL methods further improve generalizability across diseases and imaging types, reduce noise sensitivity and errors, and may enable earlier treatments and significant clinical advances [45]. While most studies remain preclinical, the evolution of automated radiographic"radiomic"markers may ultimately shift cancer diagnostics by identifying actionable tumor abnormalities [46]. Several AI models are being used for cancer detection imaging. These models include Prov-GigaPath [47], Owkin’s models [48], CHIEF [49], and Google Deepmind AI [50]. The diagnostic data sets generated also serve as valuable inputs for AI-driven drug discovery pipelines, supporting the identification of novel therapeutic candidates.

### AI for drug discovery

**Fig. 3:**
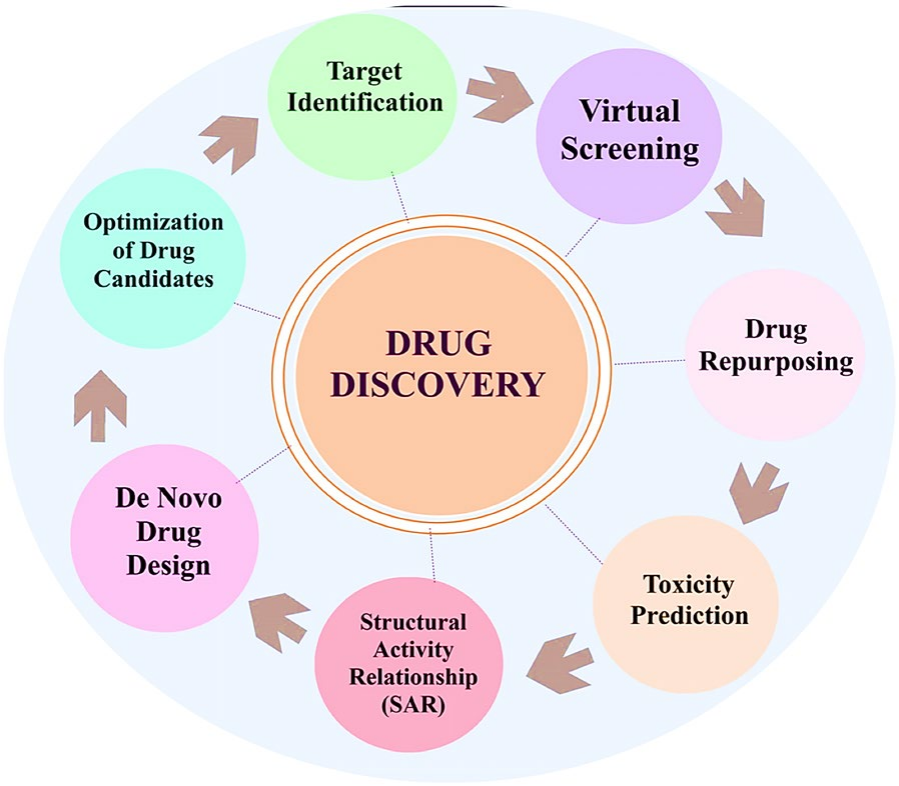
Process of drug discovery with the help of AI [11]

AI has transformed drug discovery by accelerating and enhancing multiple stages of the process. Traditional drug development is lengthy, costly, and complex, involving target identification, compound screening, lead optimization, and preclinical and clinical trials [51, 52]. AI streamlines these steps by analyzing vast biological, chemical, and clinical data sets, thereby reducing costs, shortening timelines, and improving success rates. Key applications of AI in drug discovery include target identification, compound screening, structure activity modeling, novel drug design, optimization, and repurposing, as shown in Fig. 3 [11]. Representative examples of AI tools supporting these applications are summarized in Table 1. By integrating genomics, proteomics, and molecular structure data, AI can identify disease-associated processes and promising therapeutic targets. Once identified, AI supports high-throughput virtual screening of large chemical libraries to predict binding affinities and prioritize compounds for testing [53]. ML models further analyze structure activity relationships, guiding the rational design of molecules with enhanced pharmacokinetics, specificity, and efficacy. *Zhang *et al*.* reported that AI identified a novel antibiotic candidate within weeks far faster than conventional methods by screening millions of structures against bacterial targets [54]. *Lopez *et al*.* demonstrated how AI optimizes clinical trial design by selecting patient cohorts most likely to respond to experimental therapies, thereby increasing trial success rates [55]. Beyond screening and optimization, AI generates novel drug-like molecules by learning from compound databases and experimental outcomes, thus expanding the chemical space for discovery. It also evaluates candidates using safety and ADME (absorption, distribution, metabolism, and excretion) parameters to maximize efficacy while minimizing side effects. Furthermore, AI accelerates drug repurpose by uncovering new therapeutic applications for existing compounds, reducing development risks and time to market [56]. In clinical practice, AI is increasingly integrated with robotic systems to enhance the precision of therapeutic delivery, underscoring its transformative role across the entire drug discovery and development pipeline.

**Table 1:** Examples of AI tools used in drug discovery

AI Tools	Description	Ref
DeepChem	MLP model that uses a python-based AI system to find a suitable candidate in drug discovery	[57]
DeepTox	Software that predicts the toxicity of drugs	[58]
DeepNeuralNetQSAR	Python-based system driven by computational tools that aid detection of the molecular activity of compounds	[59]
ORGANIC	A molecular generation tool that helps to create molecules with desired properties	[60]
PotentialNet	Uses NNs to predict binding affinity of ligands	[61]

### Robotics in surgery

AI’s integration into robotic surgery exemplifies its transformative influence on procedural and diagnostic precision in modern medicine [62]. Within the surgical domain, AI enhances robotic systems by delivering real time guidance and facilitating automation, thereby improving outcomes in complex surgical procedures [63]. Robotic process automation manages administrative tasks, such as billing and scheduling, freeing clinicians to prioritize patient care. *Davenport and Kalakota* showed that such automation reduces workload and burnout, improving provider well-being and care quality [30]. Simultaneously, AI demonstrates exceptional capabilities in image analysis, interpreting medical scans with a level of granularity often beyond human perception. These developments are part of a broader spectrum of AI applications that continue to redefine clinical practice [64]. Beyond initial robotic assistance, AI-driven automation in surgery contributes to greater precision and a reduction in human error. The adoption of robotic surgery is on the rise, attributed to enhanced visualization, improved dexterity, and superior ergonomic conditions for surgeons [65]. In selected surgical procedures, there is accumulating evidence supporting the non-inferiority of robotic surgery compared to laparoscopy, along with a reduction in patient morbidity [66]. Nevertheless, minimally invasive surgery remains inherently complex and technically demanding, characterized by higher variability and less favorable error profiles when contrasted with those seen in industrial settings [67]. Moreover, the introduction of new technologies into the operating room accompanied by novel technical and non-technical challenges may inadvertently increase the risk of human error and, consequently, patient harm [68]. Robotic platforms equipped with AI are designed to learn from each procedure, continuously refining surgical techniques over time and establishing new benchmarks for surgical excellence [69]. Alongside surgery, AI is also transforming pharmaceutical manufacturing by automating complex processes and ensuring product consistency.

### AI in pharmaceutical manufacturing

With the growing complexity of manufacturing processes and the increasing demand for efficiency and enhanced product quality, modern manufacturing systems are progressively aiming to transfer human expertise to machines, thereby transforming conventional manufacturing practices [70, 71]. The integration of AI into manufacturing holds substantial promise for the pharmaceutical industry. Computational tools such as computational fluid dynamics (CFD) utilize Reynolds-averaged Navier–Stokes (RANS) solvers to evaluate the effects of agitation and stress within various types of equipment, such as stirred tanks, thereby facilitating the automation of numerous pharmaceutical operations [72]. Similar approaches, including direct numerical simulations (DNS) and large eddy simulations (LES), are also employed to solve complex flow problems in manufacturing settings [73]. One notable innovation, the Chemputer platform, facilitates the digital automation of molecular synthesis and manufacturing by integrating a set of standardized chemical codes and operating through a specialized scripting language known as Chemical Assembly [59]. This platform has been successfully applied to the synthesis and production of compounds, such as sildenafil, diphenhydramine hydrochloride, and rufinamide, achieving yields and purities comparable to those obtained through manual synthesis methods [74]. In addition, AI technologies have proven effective in optimizing granulation processes in granulators ranging from 25 to 600 L in capacity [75]. These systems, utilizing techniques such as neuro-fuzzy logic, have been able to correlate critical variables with process outcomes, ultimately deriving polynomial equations to predict key operational parameters, including the proportion of granulation fluid required, impeller speed, and impeller diameter in both geometrically similar and dissimilar granulators [76]. Pharmaceutical companies are increasingly adopting AI technologies, reflecting a significant market expansion from approximately US$200 million in 2015 to US$700 million in 2018, with projections estimating growth to nearly US$5 Billion By 2024. This projected 40% increase Between 2017 and 2024 underscores AI’s potential to transform the pharmaceutical and medical sectors. Many companies have already invested heavily in AI and formed strategic collaborations to develop innovative healthcare tools. For instance, DeepMind Technologies, a subsidiary of Google, partnered with the Royal Free London NHS Foundation Trust to support the management of acute kidney injury [77, 78]. Manufacturing advances converge with AI-powered nanorobotics, enabling highly targeted drug delivery within the body.

### AI-based nanorobots for drug delivery

Nanorobots are primarily composed of integrated circuits, sensors, power supplies, and secure data backups, all maintained and managed through advanced computational technologies such as AI [79]. These nanorobots are programmed to perform a series of complex tasks, including collision avoidance, target identification, attachment to the target site, and eventual excretion from the body. Recent advances in nano and microrobotic systems have enabled navigation to specific sites within the body based on physiological cues such as pH gradients, thereby enhancing therapeutic efficacy while minimizing systemic adverse effects [80, 81]. The development of implantable nanorobots for the controlled delivery of drugs and genes necessitates careful consideration of multiple parameters, including dose regulation, sustained and controlled release mechanisms [81]. The execution of these functions relies heavily on automation, which is governed by AI-based tools, such as neural networks (NNs), fuzzy logic systems, and integrators [82]. In addition, microchip implants are employed not only for programmed drug release but also for tracking the precise location of the implant within the body [83]. In parallel, AI plays an equally critical role in rehabilitation, where robotics and data-driven systems support patient recovery.

### AI and rehabilitation

AI has introduced transformative applications in the field of rehabilitation, encompassing both physical components (e.g., robotics) and virtual systems (e.g., informatics) [84]. In rehabilitation, ML is employed in perioperative care, brain computer interfaces, myoelectric control, and symbiotic neuroprosthetics [85]. It is also applied in musculoskeletal care for analyzing patient data, supporting clinical decision-making, and interpreting diagnostic imaging. For therapeutic purposes, AI-based cognitive systems have been used to assess rehabilitation exercises based on signals from rehabilitation machines [86]. Smart home systems now assist with daily activities and alert caregivers when needed, enhancing independent living [87]. AI-enabled robotic systems could monitor and refine patient movements, aiding in the efficient execution of physical tasks during rehabilitation [88]. Robotics also play a dual role in both rehabilitation and surgery. For instance, the Hybrid Assistive Limb (HAL) exoskeleton supports patients recovering from lower limb impairments due to spinal cord injuries or strokes [89]. These systems utilize surface sensors to detect bioelectrical signals from the user’s body and convert them into coordinated joint movements [90]. Devices such as HAL and ReWalk have shown promise in restoring mobility and promoting independence in individuals with paralysis [91]. ChatGPT offers personalized and interactive support to patients, helping them stay motivated and involved throughout their rehabilitation journey, it can be configured to suggest appropriate exercises, monitor recovery milestones, and provide constructive feedback to individuals healing from physical injuries [92]. Metaverse-based neurorehabilitation combines advanced technologies, including AI-driven systems for classifying gross motor function, rehabilitation content used as motivational incentives, user-controlled virtual avatars responding to weight shifts, and deep learning-based movement evaluation. The success of these rehabilitative technologies’ mirrors AI’s growing importance in cancer care, where early detection and treatment personalization are key.

## AI in cancer

### AI for colorectal cancer

**Fig. 4:**
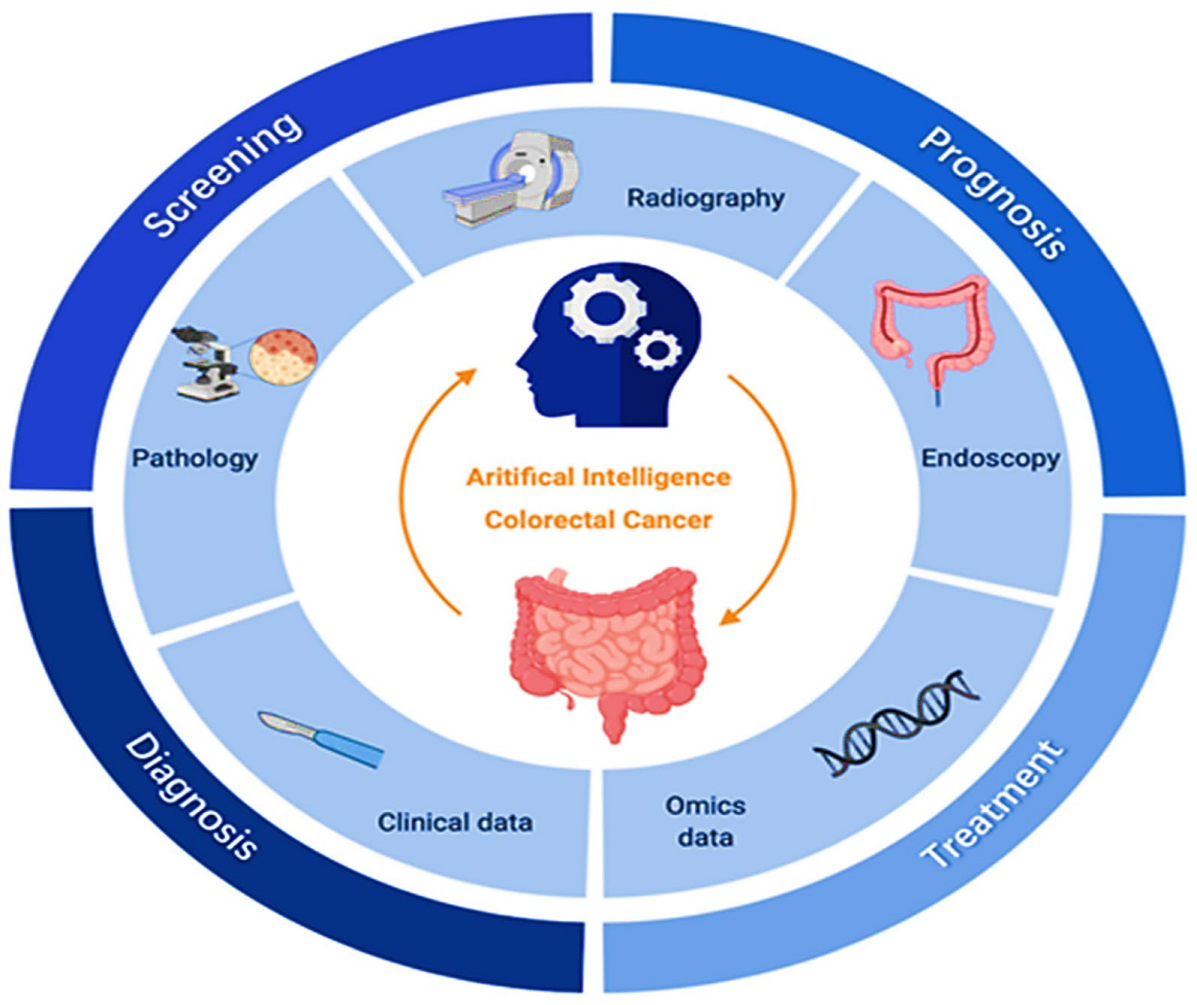
Clinical applications of AI for colorectal cancer (CRC) [93]

AI, particularly ML and DL, is transforming colorectal cancer (CRC) care by enhancing screening, diagnosis, treatment, and prognosis, as illustrated in Fig. 4 [93]. Traditional screening methods such as endoscopy and fecal occult blood tests rely heavily on clinical expertise and may miss early cases [94]. AI-assisted endoscopy improves polyp detection and characterization by analyzing large imaging data sets and electronic medical records, while predictive models using clinical and molecular data help identify high-risk individuals for earlier and more accurate screening. In diagnosis, AI-driven image recognition significantly enhances the interpretation of radiographic and pathological images for CRC staging [95]. DL algorithms reduce inter-observer variability and increase accuracy in detecting and classifying tumors from colonoscopy, biopsy, and imaging data, thereby supporting timely and precise diagnosis essential for treatment planning [96]. For treatment, AI supports optimization by predicting patient responses to surgery, chemotherapy, and radiotherapy, enabling more personalized and effective interventions. In prognosis, AI-based models integrate multidimensional clinical and molecular data to predict recurrence and estimate survival more accurately than conventional statistical methods [97]. *Yamada *et al*.* developed a real-time AI diagnostic system that detected early CRC during endoscopy with a sensitivity of 97.3%, specificity of 99.0%, and an AUC of 0.975 [98]. *Wan *et al*.* applied ML methods to whole-genome sequencing of plasma cell-free DNA for early CRC detection, analyzing gene-body Aligned reads from 546 patients with CRC and 271 controls [99]. Similarly, *Rathore *et al*.* proposed a CRC detection system based on a support vector machine radial basis function algorithm, which classified normal and malignant biopsy images and automatically determined malignancy grades [100].

#### AI in breast cancer

**Fig. 5:**
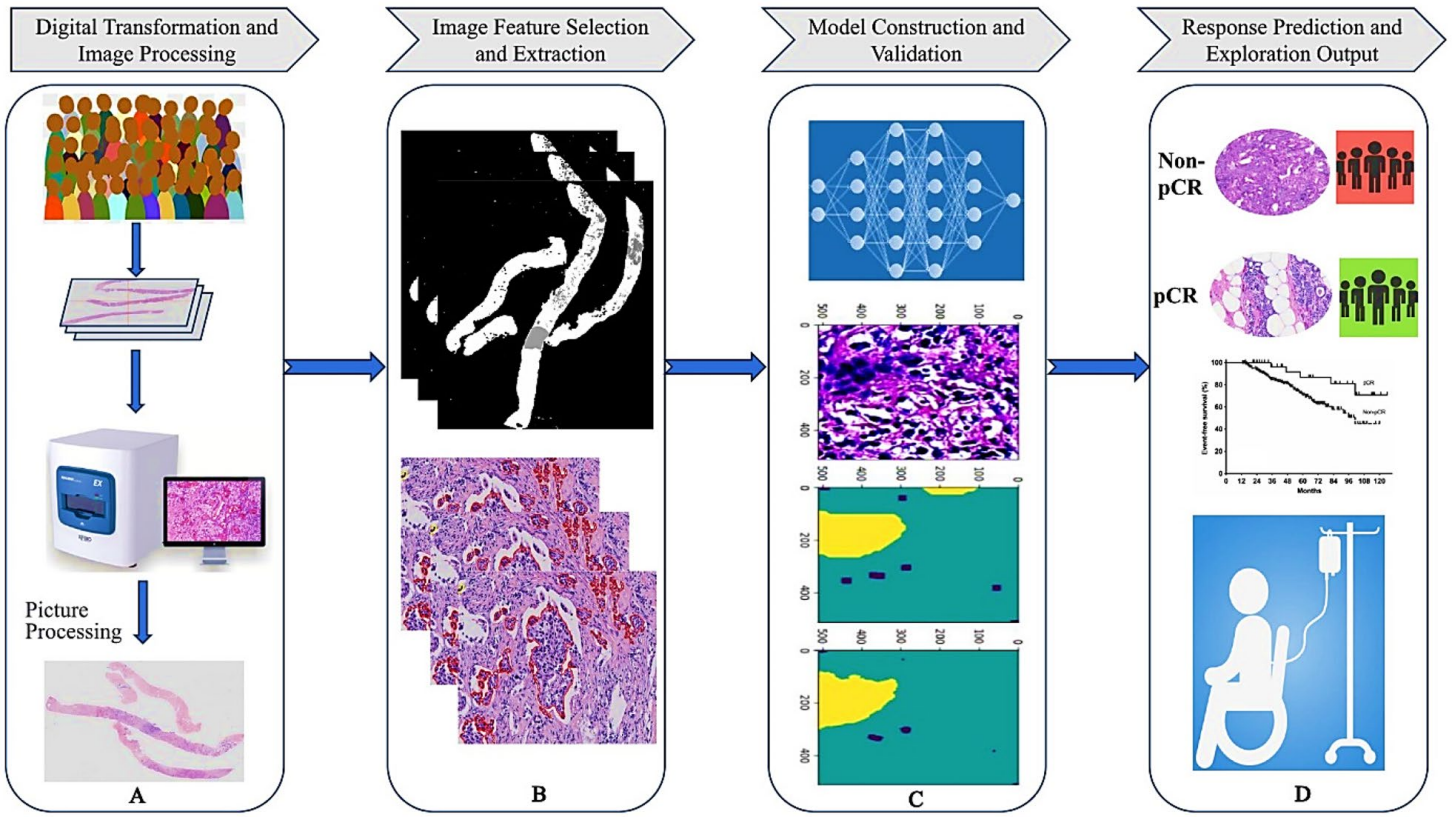
General workflow of AI-based pathology for response prediction in breast cancer: (**A**) digital transformation and preliminary image processing of pathological images; (**B**) marking, extraction, and selection of input image features; (**C**) model construction and validation; (**D**) pCR prediction and exploration of outputs [104]

Breast cancer is the most common malignant tumor in women worldwide [101]. Neoadjuvant therapy (NAT) can improve treatment outcomes, but patient responses vary considerably [102]. Conventional approaches for evaluating NAT response, such as histopathology and biomarker assessment, are limited in accuracy and efficiency [103]. AI has advanced the prediction of NAT efficacy by integrating digital pathology with computational models, allowing individualized evaluation before systemic treatment, as shown in Fig. 5 [104]. Pathomics extends beyond traditional H&E staining by incorporating molecular markers (ER, PR, HER2, Ki67, and PD-L1) along with genomic and proteomic data that reflect tumor sensitivity to therapies. By merging these diverse features with AI, researchers can more accurately predict responses to neoadjuvant regimens in breast cancer [104]. Several AI models demonstrate these applications such as *Cruz-Roa *et al*.* who developed a convolutional neural network (CNN) that classified invasive ductal carcinoma patches from whole-slide images (WSI) and estimated infiltration using a ConvNet classifier [105]. While *Han *et al*.* reported DL model with an average accuracy of 93.2% across eight classes (four benign and four malignant) [106]. *Luo *et al*.* proposed a deep learning-based clinical risk stratification model for overall survival in young women with breast cancer, integrating histological features with clinical data to outperform conventional prognostic tools [107]. Similarly, *Huang *et al*.* developed a model to improve histological grading and predict upstaging of atypical ductal hyperplasia and ductal carcinoma in situ from biopsies [108]. In addition, AI models analyzing pre- and post-treatment imaging features help anticipate response to neoadjuvant chemotherapy, recurrence risk, and survival outcomes. This predictive capability enables clinicians to tailor therapy according to tumor biology and expected treatment sensitivity, supporting the transition toward more precise and personalized breast cancer care.

#### AI in lung cancer

**Fig. 6:**
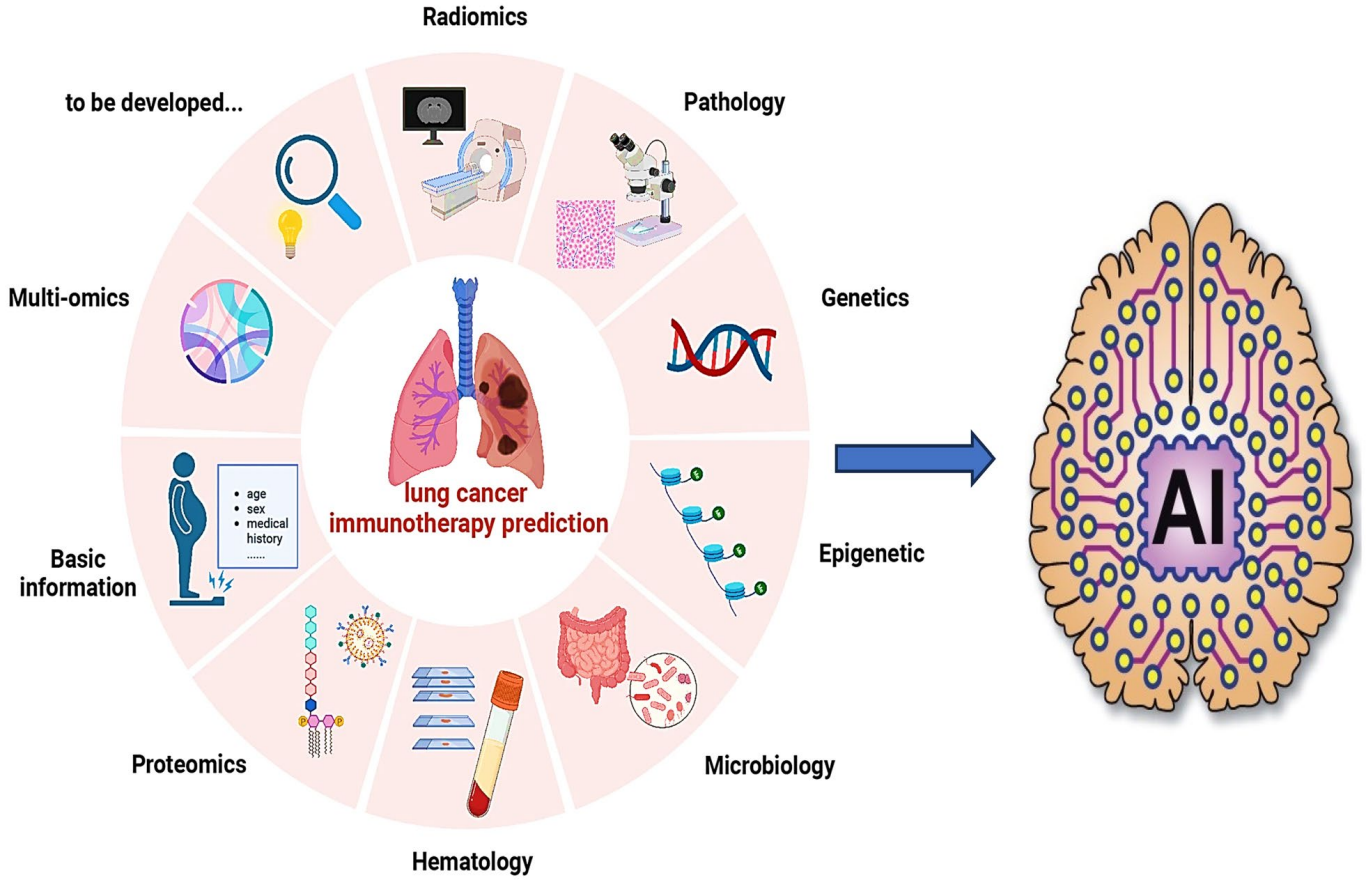
AI-based technologies in lung cancer immunotherapy [112]

Lung cancer is one of the most common malignancies worldwide and remains the leading cause of cancer-related mortality [109]. Immune checkpoint inhibitors (ICIs) targeting PD-1, PD-L1, and CTLA-4 have demonstrated significant efficacy in the treatment of non-small cell lung cancer (NSCLC). However, only about 30% of patients are eligible for these therapies, and immune-related adverse events remain a clinical challenge [110, 111]. Traditional evaluation methods are often insufficient for predicting therapeutic benefit, highlighting the need for more advanced approaches. AI-based technologies addressing these challenges in lung cancer immunotherapy are summarized in Fig. 6 [112]. AI has emerged as a powerful tool in lung cancer care, contributing to early detection, diagnosis, and treatment optimization. ML model can distinguish benign from malignant nodules, monitor tumor growth, and enhance bronchoscopic procedures by improving diagnostic accuracy and lymph node sampling yield [113]. The prediction of therapy efficacy can be classified into direct predictions and indirect predictions. Common approaches such as radiomics, pathomics, and genomics can indirectly predict the relationship between PD-L1, TMB, and other biomarkers with survival and therapy efficacy. Conversely, proteomics and laboratory inspection data are mainly utilized for direct predictions. In pathology, AI automates tissue analysis, improving diagnostic accuracy, classification of lung cancer subtypes, and prognostic assessment [114]. Together, these applications support precision oncology by enabling individualized, data-driven interventions that improve survival outcomes. A predictive model for lymph vascular invasion (LVI) and nodal involvement achieved a sensitivity of 75.8%, specificity of 67.6%, accuracy of 70.8%, and an AUC of 0.77 [100]. *Zhong *et al*.* developed a DL model using chest CT images from 3,096 patients with stage I NSCLC, achieving an AUC of 0.82 for predicting N2 metastasis and enabling prognostic stratification [115]. *Yan’s* team trained a DL-based detection model on the LUNA16 public database and validated it on the Anti-PD-1_Lung data set, demonstrating the ability to predict immunotherapy response [110]. Similarly, *Mu *et al*.* extracted features from PET–CT images obtained before ICI treatment and developed a predictive model for overall survival (OS) and progression-free survival (PFS) [116]. Beyond cancer care, AI applications are also reshaping patient engagement and chronic disease management, particularly through the development of virtual health assistants that provide continuous monitoring, personalized guidance, and decision support.

### Development of virtual health assistants

Virtual Health Assistants (VHAs) are broadly defined as AI-driven platforms that interact with patients to provide health-related information, reminders, or support. The development of VHAs has transformed patient engagement and healthcare accessibility through the integration of advanced AI technologies, particularly NLP and conversational AI [117]. Within this category, *chatbots* represent a specific subset focused primarily on text- or voice-based conversational exchanges, whereas more advanced VHAs may include multimodal systems such as voice-activated agents and relational agents designed to establish trust and rapport with users. Early chatbot implementations such as the Ada Health app employ machine learning to assess symptoms and suggest potential diagnoses, with studies showing Alignment with physician recommendations in more than 70% of cases [118]. In contrast, voice-based VHAs like Amazon Alexa with healthcare functionalities assist elderly patients in managing chronic conditions, while relational agents explored by *Bickmore *et al*.* are designed to simulate human-like interaction, thereby improving user engagement and long-term adherence [119]. *De Choudhury *et al*.* showed that conversational agents can analyze linguistic patterns to detect early signs of depression or anxiety, enabling timely interventions [120]. Similarly, *Miner *et al*.* highlighted their potential to support acute psychological needs, such as guiding patients through relaxation techniques during panic attacks [121]. Despite these benefits, adoption rates remain uneven and are influenced by factors, such as age, digital literacy, cultural perceptions, and levels of trust in AI systems. Patients often express greater willingness to use VHAs when transparency, data privacy, and human oversight are emphasized, while skepticism persists regarding their clinical reliability and ethical use. Current systems may misinterpret ambiguous or context-specific language, struggle with complex medical terminology, or fail to recognize cultural nuances, which can undermine trust and safety. Cost-effective assessments are necessary to ensure sustainable implementation, as the development and deployment of VHAs can involve substantial financial investment.

### AI integration with wearable devices

Modern wearable health technologies have progressed from simple biometric trackers to intelligent monitoring systems through the integration of AI [122]. These devices now process continuous streams of physiological data to detect health patterns, predict risks, and deliver personalized recommendations, establishing a proactive, data-driven model of wellness management. They capture key health metrics including cardiovascular function, metabolic indicators, and behavioral patterns generating longitudinal data sets essential for identifying trends and enabling timely interventions [123]. Research highlights their particular value in chronic disease management, where real-time physiological monitoring enhances clinical decision-making [124]. AI transforms wearables from passive data collectors into active diagnostic assistants through advanced signal processing and pattern recognition. ML architectures extract clinically meaningful insights from biometric streams, while DL models detect subtle pathological signatures, such as arrhythmias in ECG waveforms or early hypertension indicators in blood pressure trends. Wearable technologies include smartwatches, fitness trackers, and medical-grade sensors capable of monitoring parameters, such as heart rate, blood pressure, blood glucose, sleep quality, and physical activity [125]. In diabetes care, AI-enhanced continuous glucose monitors provide real-time glycemic feedback and analyze behavioral data to optimize diet and activity, representing a major shift in self-management [126]. The integration of advanced sensors with ML promises even deeper insights into health, positioning wearables as central tools in the future of personalized healthcare delivery. Building on these examples of AI-enabled personalized healthcare tools, Table 2 highlights landmark studies and clinically deployed AI systems across multiple medical domains, illustrating how AI translates from research into practical, real-world applications.

**Table 2:** Different applications of AI in healthcare and pharmaceutical industry

Application	AI Tool	Contribution	Ref
Diabetic Retinopathy Detection	Dx-DR, EyeArt, and AEYE-DS	FDA-approved autonomous AI system for diagnosing diabetic retinopathy	[127]
Breast Cancer Detection	LYmph Node Assistant or LYNA	Deep learning algorithm that significantly improves pathologists'sensitivity in detecting metastatic breast cancer in sentinel lymph node biopsies	[128]
Robotic Surgery Assistance	da Vinci Surgical System	Advanced robotic platform enabling minimally invasive surgeries with enhanced precision and control	[129]
Brain tumour	VGG16 architecture	VGG16 architecture for medical image classification, specifically in brain tumour and Alzheimer data set	[130]
Sepsis Early Warning	Epic Sepsis Model	Predictive analytics tool integrated into electronic medical records to identify sepsis onset earlier than traditional methods	[131]
Chest CT	AI-Rad Companion (Siemens Healthineers®)	Analyzing chest CT scans and comparing the results against Radiologists’ evaluation	[132]
Drug Discovery	AlphaFold (DeepMind)	AI system capable of predicting protein structures with high accuracy, accelerating drug discovery processes	[133]
Brain Tumor Segmentation	DeepMedic	Deep learning-based method for automatic brain tumor segmentation in MRI scans, aiding neurosurgery planning	[134]
Personalized Medicine	IBM Watson for Oncology	AI-driven clinical decision support system providing cancer treatment recommendations based on patient data	[135]
Lung Nodule Detection	InferRead CT Lung	AI algorithms that automatically analyze medical images to detect lung nodules	[136]

## Challenges and existing solutions

### Data integration and interoperability

#### Challenges

In smart healthcare, data heterogeneity hinders AI adoption, because variations in structures, formats, and standards complicate integration across departments, institutions, and medical systems. This challenge spans structured, semi-structured, and unstructured data, as well as diverse modalities, such as text, images, audio, and video, each requiring specialized processing. Even within similar data types, inconsistencies like differing disease coding systems add complexity. Addressing these issues requires unified standards, robust cross-system conversion tools, and advanced machine learning methods to harmonize data sets and support reliable, scalable AI applications [137].

#### Existing solutions

Several strategies support data integration and interoperability in healthcare. The Artificial Intelligence Modern Data Platform (AIMDP) manages both structured and unstructured information such as lab results, monitoring data, and clinical notes to generate insights that guide treatment decisions [138]. Data harmonization pipelines convert diverse data sets into unified formats using the FHIR standard, ensuring consistency and usability for AI applications, such as integrating blood glucose, weight, and dietary records in diabetes management. Direct adoption of Health Level 7 FHIR standards further enables seamless data modeling and exchange across institutions, allowing oncology specialists in different hospitals to access shared genetic, treatment, and clinical information for coordinated, personalized cancer care [139, 140].

### Large-scale data handling

#### Challenges

Healthcare is experiencing unprecedented data growth from electronic health records (EHRs), wearable devices, Internet of Things (IoT) technologies, imaging, clinical notes, and genomics [141]. The scale, diversity, and velocity of these data sets often exceed the capabilities of traditional data management systems, creating difficulties in storage, processing, and integration. Radiology departments, for instance, generate terabytes of imaging data annually, while EHRs capture detailed patient histories and treatment outcomes. AI provides powerful tools for analysis, its success relies heavily on advances in underlying data infrastructure [142]. Without scalable platforms, efficient analytics, and robust governance, the potential of AI in healthcare remains limited.

#### Existing solutions

To address these challenges, several approaches have been proposed, including ML, agent-based, heuristic, cloud-based, and hybrid mechanisms, though each poses trade-offs in resource use, privacy, and complexity [143]. Distributed frameworks, such as Hadoop, using MapReduce, have been applied in projects like the Mayo Clinic to aggregate large-scale EHRs [144]. Apache Spark provides in-memory processing that accelerates genomic analyses in initiatives such as Genomics England, though it demands significant resources [145]. Data mining techniques also support predictive modeling, as demonstrated in the Diabetes Control and Complications Trial (DCCT), where decision trees and neural networks were applied to assess health risks and guide personalized care.

### Real-time processing

#### Challenges

In smart healthcare, the rapid growth of IoT devices has generated vast amounts of data requiring real-time processing, which traditional cloud-based methods struggle to handle due to latency issues. To address this, fog and edge computing has emerged as effective solutions by decentralizing data processing and bringing computation closer to the data source. Edge computing enables immediate analytics at the device level, such as wearable monitors analyzing patient vitals in real time. In contrast, fog computing provides additional storage and processing at intermediate nodes, supporting larger tasks like integrating data across a hospital network. Together, these approaches reduce latency, improve efficiency, and enhance the responsiveness of intelligent healthcare systems [146].

#### Existing solutions

To overcome these challenges, decentralized computing approaches such as edge and fog computing have been developed. Edge computing enables real-time analytics at or near devices, as in wearable monitors that process patient vitals locally, while AI tasks can be divided between reasoning at the edge and training in the cloud [147]. Advanced frameworks like Smart-Edge-CoCaCo further improve efficiency by coordinating communication, caching, and computation [148]. Fog computing, positioned between edge and cloud, provides greater storage and processing capacity, making it suitable for hospital-wide data integration. When combined with DL, fog systems have been applied in intelligent medical monitoring, where physiological data are processed efficiently to deliver timely and accurate health insights.

### Model interpretability

#### Challenges

A major challenge in healthcare AI is the opacity of ML and DL models, which often operate as “black boxes” that provide predictions without revealing the reasoning behind them. This lack of transparency undermines clinical trust, complicates validation of AI-driven diagnoses, and limits patient confidence in their care. For example, an AI system may suggest a diagnosis without clarifying which features influenced the result, reducing its reliability in evidence-based practice. To address this, interpretable models are needed to clarify decision-making processes, highlight key features, and foster trust among clinicians and patients [149].

#### Existing solutions

To overcome these challenges, Explainable AI (XAI) techniques have been developed to reveal how models generate predictions by identifying feature importance, correlations, and reasoning pathways [150]. Common methods include LIME, which approximates local model behavior; SHAP, which quantifies feature contributions; Grad-CAM, which highlights image regions influencing predictions; and t-SNE, which visualizes high-dimensional data. These tools are increasingly applied in healthcare for instance, Grad-CAM has been used to locate areas of concern in retinal images for diabetic retinopathy diagnosis, while SHAP helps interpret factors contributing to patient readmission risks. By improving transparency and accountability, XAI enhances the usability, reliability, and acceptance of AI systems in clinical practice [151].

### Continuous learning and adaptability

#### Challenges

AI in healthcare faces difficulties in continuous learning, as medical knowledge, treatments, and practices evolve rapidly. Models trained on static data sets risk becoming outdated, leading to declining accuracy over time [152]. The COVID-19 pandemic highlighted this limitation, as diagnostic tools needed rapid adaptation to emerging variants and shifting clinical protocols. Similarly, advances such as CAR-T therapies and robot-assisted surgeries require AI systems to incorporate new evidence to remain clinically relevant. Examples like IBM Watson Health, which updates its knowledge base with current research and trial data, underscore the importance of regular model refinement to sustain accuracy, effectiveness, and trustworthiness [153].

#### Existing solutions

To overcome these challenges, continuous learning techniques have been introduced to enable AI models to adapt to evolving data and practices [154]. Applications include Dexcom’s glucose monitors adjusting insulin doses in real time, BlueDot issuing early outbreak alerts during COVID-19, and Tempus Labs refining genomic-based therapies. A key obstacle is catastrophic forgetting, where new knowledge disrupts previously learned information. Strategies to address this include regularization, replay, optimization, representation learning, and architecture-based methods [155]. Practical implementations, such as pairing a k-NN classifier with a fixed pre-trained feature extractor, help maintain adaptability while controlling computational and storage demands, ensuring AI systems remain reliable in dynamic healthcare environments [156].

### Security of AI models

#### Challenges

AI in healthcare faces critical security threats across all stages of operation, from data collection to preprocessing, training, and inference. Sensitive medical data are exposed to risks such as sensor spoofing during acquisition, scaling attacks during preprocessing, and adversarial manipulations that subtly alter inputs to trigger incorrect predictions or compromise privacy. These vulnerabilities undermine both patient safety and system integrity. Protecting data sources from tampering and enhancing model robustness against adversarial attacks are, therefore, essential to building secure and trustworthy AI-driven healthcare systems [157].

#### Existing solutions

To overcome these challenges, multiple defensive strategies have been developed. Remote monitoring systems, such as glucose and heart-rate sensors, employ anomaly detection and data fusion with EHRs to identify falsified signals. In medical imaging, robust scaling algorithms and median filters preserve diagnostic accuracy by correcting artifacts and restoring image quality. To counter adversarial attacks, techniques such as adversarial retraining, data cleaning, and input reconstruction improve model resilience, while generative adversarial networks (GANs) are used to simulate attacks and generate high-quality training images that strengthen predictive performance [158]. Collectively, these approaches enhance data integrity, diagnostic precision, and the overall robustness of healthcare AI systems.

### Ethical AI design

#### Challenges

Ensuring ethical AI in healthcare requires integrating fairness, safety, privacy, and accountability into systems from the design stage rather than as afterthoughts [159]. A major challenge is determining responsibility when errors occur, as opaque model decisions make it difficult to attribute liability between clinicians, developers, and institutions. Bias also poses a significant risk, often stemming from imbalanced data sets or flawed design, which can reinforce healthcare inequalities. Furthermore, managing sensitive patient data raises privacy concerns, where breaches threaten both ethical and legal standards [160]. These challenges underscore the need for fairness, transparency, and accountability in building trustworthy AI systems.

#### Existing solutions

To overcome these challenges, ethical AI frameworks emphasize accountability, fairness, and privacy. Clear responsibility guidelines are needed to define the roles of developers, clinicians, and users in cases of errors. Bias mitigation strategies include using diverse data sets, conducting fairness audits, validating models across populations, and educating stakeholders, with combined approaches offering the most effective outcomes [161]. Patient privacy can be safeguarded through encryption, anonymization, and differential privacy, while advanced techniques such as federated learning enable collaborative model training without sharing raw data. Homomorphic encryption further allows computations on encrypted data sets, supporting secure data use. Collectively, these approaches provide practical pathways toward transparent, fair, and privacy-preserving AI in healthcare [162].

### Scalability

#### Challenges

Scalability remains a significant challenge in deploying AI in healthcare, as models that perform well in small-scale trials often struggle to maintain accuracy, speed, and integration when applied across large national systems. The vast volumes of patient data, diverse medical conditions, and the need for compatibility with different healthcare IT infrastructures complicate large-scale implementation. Addressing this requires advanced data processing strategies and robust system architectures, as demonstrated by the Mayo Clinic, which enhanced its processing modules to manage nationwide medical data efficiently, thereby improving diagnostic accuracy, response time, and overall system reliability [163].

#### Existing solutions

Modular architecture and cloud computing are widely adopted solutions to achieve scalability in healthcare AI. As demonstrated by Mount Sinai's Modular Health Information System, modular systems divide applications into independent, task-specific units that can operate concurrently, enhancing speed, efficiency, and adaptability. Cloud platforms such as Google Cloud and Amazon Web Services (AWS) support scalability by dynamically adjusting computational and storage resources to meet workload demands [164]. This enables efficient Handling of large medical data sets while ensuring security and reliability. In addition, new Benchmarking methodologies such as BigDataBench 4.0 and Mystique have emerged to replace traditional, non-scalable approaches, offering realistic frameworks for evaluating the performance of big data and AI systems in healthcare contexts [165].

### Underserved and remote areas with limited connectivity

#### Challenges

Deploying AI in resource-limited and remote healthcare settings faces major hurdles due to unstable or absent internet connectivity, which restricts real-time analytics, data uploading, and system updates. To address these challenges, offline AI models and portable devices have been developed to function independently of continuous network access [166]. For example, GE Healthcare’s portable ultrasound systems can perform local image analysis and store results for later upload, enabling immediate diagnoses even in low-connectivity regions. Such offline AI solutions ensure reliable diagnostic support and make healthcare more accessible in resource-constrained environments [167].

#### Existing solutions

Several strategies can complement edge and fog computing to enhance the deployment of AI in resource-limited healthcare environments. Offline AI models allow devices like GE Healthcare’s portable ultrasound systems to provide immediate diagnoses without internet access by storing results locally [168]. Data compression techniques, such as Gzip and Brotli, used in wearable devices like Fitbit, reduce transmission demands in low-bandwidth settings [169]. Lightweight AI models, including MobileNet and TinyYOLO, enable real-time analysis on low-power devices, with Huawei’s Atlas 200 accelerator card exemplifying their effectiveness. In addition, low-bandwidth optimization methods, such as adaptive compression algorithms employed in drone-based disaster relief, improve data transfer efficiency under unstable network conditions [163]. These approaches enhance the accessibility, reliability, and resilience of AI systems in underserved and remote healthcare settings, helping to bridge the gap between advanced digital technologies and real-world clinical needs.

## Bias in healthcare AI and mitigation strategies

### Sources of bias

#### Data biases

Most areas of human research remain heavily biased toward participants with a Western, Educated, Industrialized, Rich, and Democratic (WEIRD) profile [170], making them unrepresentative of the global population. Since many data sets used to train AI are derived from such studies, these biases are inherited by algorithms. Some data set biases, like ethnicity in skin images or gender in genetics, are easy to detect, while others, such as socioeconomic status or sexual orientation, require explicit metadata. Even seemingly unrelated metadata can be crucial for identifying bias [171]. For instance, neuroscience research has shown that socioeconomic variables correlate with detectable differences in brain structure and function [172]. To properly evaluate such influences, future studies must incorporate standardized metadata on factors that may introduce bias.

#### Algorithmic biases

When algorithms are trained on Biased data sets, they tend to reinforce patterns from the dominant class. For example, if a data set contains 80% Healthy and 20% diseased images, an algorithm could achieve 80% accuracy simply by labeling all samples as healthy. To prevent misinterpretation, it is essential to establish objective estimates of chance performance. One approach is permuting sample labels and retraining the algorithm to generate “random” predictions, providing an empirical baseline for chance levels [173]. This should be complemented with performance metrics robust to class imbalance or classification methods that incorporate weighting factors during optimization to account for under-represented classes [174].

#### Clinician interaction-related biases and resistance

The adoption of AI in healthcare relies on clinician trust, yet resistance persists due to concerns over reliability, workflow disruption, and medical mistrust shaped by historical disparities. *Alsheibani *et al*.*, using the technology–organization–environment (TOE) framework, identified organizational, technological, and environmental barriers to AI adoption and stressed leadership’s role in overcoming them [175]. In addition, *Lee and Rich* emphasized perceptual and social factors, highlighting how historical mistrust shapes clinicians’ acceptance of AI [176]. While *Strohm *et al*.* found that unclear integration processes, variable trust, and uncertain clinical value limit adoption in radiology [177]. *Cadario *et al. reported that insufficient understanding of AI algorithms and blurred decision-making roles drive resistance, recommending targeted education to strengthen engagement [178]. Clinician resistance is reinforced by biased AI systems, making diverse data, regular audits, and continuous validation essential to build trust and ensure equitable use.

#### Patient interaction-related biases

Patient-related biases involve disparities in access and interaction with AI systems. Privilege bias arises when certain populations lack access to AI-enabled care or the necessary technology, reinforcing existing inequities [179]. Informed mistrust reflects skepticism rooted in historical healthcare injustices, leading some to avoid care or conceal information. Agency bias results from patients’ limited roles in AI development and evaluation, meaning their needs and perspectives may be inadequately represented in AI-driven healthcare [180].

### Strategies to mitigate bias

#### Data diversity and validation

Ensuring AI fairness starts with collecting and using diverse, representative data sets that reflect the full spectrum of patient demographics, conditions, and healthcare settings. This approach reduces biases and improves generalizability. Regular audits and independent validations by experts help detect and correct emerging biases. Healthcare institutions should implement continuous AI performance monitoring systems to adapt models over time as clinical environments evolve [181].

#### Education and awareness

Educating clinicians and patients about AI biases fosters critical use and informed decision-making. Clinicians trained on potential biases avoid over reliance and can better evaluate AI outputs, while informed patients engage more effectively in their care discussions. Promoting collaborative communication channels such as workshops and forums supports ongoing learning, user feedback, and iterative improvements to AI systems [182].

#### Ethical and legal frameworks

Robust ethical and legal structures are essential for protecting patient privacy, ensuring data security, and defining clear accountability for AI-driven decisions [7]. Obtaining informed consent and complying with regulations, such as HIPAA and GDPR safeguard data use. Clear liability frameworks allocate responsibility among clinicians, developers, and institutions. Enhancing algorithm explainability promotes transparency and trust, though awareness of explainability’s limitations is important to mitigate confirmation biases [183].

#### Stakeholder collaboration

Mitigating AI biases demands coordinated efforts from a wide range of stakeholders. Physicians provide essential clinical expertise, AI developers refine and optimize algorithms, and policymakers establish clear regulatory frameworks [159]. Patients and advocacy groups bring forward community perspectives and equity concerns, while professional associations define ethical standards. Such multidisciplinary collaboration is crucial to ensure that AI technologies are designed and implemented in a responsible, fair, and effective manner across healthcare systems [184].

## Future perspectives and recommendations

The future of AI in healthcare will be driven by advances in computational power, algorithmic innovation, and the growing availability of multimodal data sets, including medical imaging, genomics, proteomics, metabolomics, and electronic health records. Integrating these data streams will enable highly precise diagnoses, personalized treatment planning, and dynamic, adaptive patient care. Future systems may integrate multi-omics data sets to model disease progression and simulate treatment outcomes through “digital twins” virtual patient replicas [185]. In drug discovery, advanced AI models, including quantum-enhanced systems, are being developed to simulate molecular behavior, predict off-target effects, and streamline compound screening [186]. In clinical practice, the evolution of explainable AI frameworks will be critical to ensure transparency, interpretability, and trust among healthcare providers. AI is also expected to reshape preventive medicine by identifying at-risk populations through predictive modeling and enabling early, personalized interventions. In oncology, the future lies in deeper AI-driven analyses of the tumor microenvironment, facilitating more individualized and adaptive immunotherapies.

Looking ahead, AI will not only optimize clinical workflows but also reshape healthcare delivery systems on a global scale. Lightweight and mobile-compatible AI solutions designed for low- and middle-income countries (LMICs) could reduce disparities in access to high-quality care, provided infrastructure and capacity-building needs are met. Global collaborations, open-access data initiatives, and coordinated funding mechanisms will be central to ensuring that innovation is both equitable and sustainable. However, to realize these opportunities, several enabling conditions must be addressed, encompassing data diversity, regulatory frameworks, cost-effectiveness, and collaborative governance.

First, research must prioritize the creation of diverse, validated, and externally tested models to ensure fairness and reliability across populations and health systems. Initiatives such as the UK Biobank and the National COVID Cohort Collaborative (N3C) exemplify efforts to promote data diversity, yet comparable frameworks remain limited in many regions. The lack of standardized, multiethnic data sets poses a major challenge, as biased training data can perpetuate inequities in diagnosis and treatment. The associated risk of non-generalizable or discriminatory outcomes can be mitigated by mandating independent benchmarking data sets, promoting international data-sharing under secure privacy-preserving conditions, and incorporating fairness audits into the development pipeline.

Second, policy must establish adaptive ethical and regulatory frameworks that balance innovation with accountability and transparency. Notable efforts include the U.S. FDA’s AI/ML-based SaMD Action Plan, the EU AI Act, and the World Health Organization’s guidance on ethics and governance of AI for health [187]. Despite these advances, many countries lack clear or enforceable guidelines, and the dynamic nature of learning systems complicates regulatory oversight. The risk of fragmented standards or regulatory lag can hinder both innovation and patient safety. To mitigate this, international harmonization platforms and regulatory sandboxes should be established to test emerging technologies under controlled conditions, ensuring safety while facilitating continuous adaptation [188].

Third, successful implementation will depend on prioritizing cost-effective, resource-efficient AI tools alongside workforce training and digital literacy. In LMICs, initiatives such as AI4Health Africa and PATH’s Digital Square demonstrate the feasibility of low-cost, scalable AI applications, yet challenges persist in terms of infrastructure limitations, workforce capacity, and sustainable funding. Without targeted support, there is a risk of exacerbating the digital divide, leaving resource-limited settings further behind. This can be mitigated through blended investment models that combine public–private partnerships, international aid, and capacity-building programs to strengthen infrastructure while simultaneously training healthcare professionals in AI literacy and clinical integration [189, 190].

Finally, global collaboration will be essential to enable scalable and equitable deployment of AI in healthcare. Initiatives such as the Global Alliance for Genomics and Health (GA4GH) and the International Cancer Genome Consortium highlight the value of international data-sharing, but significant barriers remain, including data privacy concerns, interoperability issues, and geopolitical restrictions. The risk of data silos can limit generalizability and innovation. Federated learning and privacy-preserving computation offer mitigation strategies by allowing models to be trained on distributed data without compromising patient confidentiality. Furthermore, international agreements on data governance and equitable benefit-sharing will be essential to overcome geopolitical divides and ensure global access to AI-driven advances [191].

In summary, the transformative potential of AI in healthcare is undeniable, but its realization will depend on addressing critical enablers and barriers. By grounding future developments in diverse data sets, adaptive policies, cost-effective implementation strategies, and international collaboration, AI can move from experimental promise to sustainable integration in clinical practice. At the same time, explicit recognition of the risk’s bias, regulatory gaps, inequitable access, and data fragmentation together with proactive mitigation strategies will ensure that AI evolves as a safe, ethical, and globally accessible tool for improving human health.

## Conclusion

Artificial intelligence is no longer a distant prospect but an integral component of modern healthcare, transforming diagnostics, drug discovery, precision medicine, and health system operations. However, for AI to progress from promising innovations to globally trusted solutions, stakeholders must go beyond proof-of-concept applications and focus on scalable, equity-driven implementation. Future success will depend on three critical priorities: developing algorithms trained on diverse and representative data sets; embedding cost-effectiveness and sustainability analyses into deployment strategies; and establishing global regulatory frameworks that ensure transparency, ethical responsibility, and patient safety. Equally important is the translation of AI tools into real-world clinical settings, particularly in low- and middle-income countries, through lightweight, resource-efficient models that address infrastructure gaps. Building clinician capacity through AI literacy and training will be essential for ensuring human oversight in decision-making. By aligning technological innovation with practical implementation, policy development, and global collaboration, AI can transition from incremental improvements to a reliable, equitable, and sustainable foundation for healthcare worldwide.

## Data Availability

No datasets were generated or analysed during the current study.

## References

[1] Yeganeh H. An analysis of emerging trends and transformations in global healthcare. Int J Health Gov. 2019;24(2):169–80.

[2] Tabish SA, Nabil S. Future of healthcare delivery: strategies that will reshape the healthcare industry landscape. Int J Sci Res. 2015;4(2):727–58.

[3] Sheth A, Roy K, Gaur M. Neurosymbolic artificial intelligence (why, what, and how). IEEE Intell Syst. 2023;38(3):56–62.

[4] Suriyaamporn P, et al. The artificial intelligence-powered new era in pharmaceutical research and development: a review. AAPS PharmSciTech. 2024;25(6):188. 39147952 10.1208/s12249-024-02901-y

[5] Patil S, Shankar H. Transforming healthcare: harnessing the power of AI in the modern era. Int J Multidiscip Sci Arts. 2023;2(2):60–70.

[6] Ahmed Z, et al. Artificial intelligence with multi-functional machine learning platform development for better healthcare and precision medicine. Database. 2020;2020:baaaa010. 10.1093/database/baaa010 PMC7078068 32185396

[7] Shoghli A, Darvish M, Sadeghian Y. Balancing innovation and privacy: ethical challenges in AI-driven healthcare. J Rev Med Sci. 2024;4(1):1–11.

[8] Alowais SA, et al. Revolutionizing healthcare: the role of artificial intelligence in clinical practice. BMC Med Educ. 2023;23(1):689. 37740191 10.1186/s12909-023-04698-z PMC10517477

[9] Edison G. Transforming medical decision-making: A comprehensive review of AI’s impact on diagnostics and treatment. BULLET. 2023;2(4):1121–33.

[10] Khalid H, et al. A comparative systematic literature review on knee bone reports from MRI, x-rays and CT scans using deep learning and machine learning methodologies. Diagnostics. 2020;10(8):518. 32722605 10.3390/diagnostics10080518 PMC7460189

[11] Kumar P, et al. Advanced artificial intelligence technologies transforming contemporary pharmaceutical research. Bioengineering. 2025;12(4):363. 40281723 10.3390/bioengineering12040363 PMC12024664

[12] Taye MM. Understanding of machine learning with deep learning: architectures, workflow, applications and future directions. Computers. 2023;12(5):91.

[13] Sarker M. Revolutionizing healthcare the role of machine learning in the health sector. J Artif Intell General Sci (JAIGS) ISSN. 2024;2(1):36–61.

[14] Karalis VD. The integration of artificial intelligence into clinical practice. Appl Biosci. 2024;3(1):14–44.

[15] Miotto R, et al. Deep learning for healthcare: review, opportunities and challenges. Brief Bioinform. 2018;19(6):1236–46. 28481991 10.1093/bib/bbx044 PMC6455466

[16] Sarvamangala DR, Kulkarni RV. Convolutional neural networks in medical image understanding: a survey. Evol Intell. 2022;15(1):1–22. 33425040 10.1007/s12065-020-00540-3 PMC7778711

[17] Mienye ID, Swart TG, Obaido G. Recurrent neural networks: a comprehensive review of architectures, variants, and applications. Information. 2024;15(9):517.

[18] Derek, V. and P. Collings, Natural Language Processing (NLP) in Healthcare AI: Enhancing Clinical Insight Extraction from Unstructured Patient Data. 2025.

[19] Sarella PNK, Mangam VT. Ai-driven natural language processing in healthcare: transforming patient-provider communication. Indian J Pharm Pract. 2024. 10.5530/ijopp.17.1.4.

[20] Singla, S., et al. Advancements in Natural Language Processing: BERT and Transformer-Based Models for Text Understanding. IEEE.

[21] Pramanik B, et al. Beyond prediction: how generative AI is creating new healthcare realities. In: Revolutionizing Healthcare 50 the power of generative AI advancements in patient care through generative AI algorithms. Cham: Springer; 2025.

[22] Lang O, et al. Using generative AI to investigate medical imagery models and datasets. EBioMedicine. 2024;1:102. 10.1016/j.ebiom.2024.105075 PMC10993140 38565004

[23] Zeng X, et al. Deep generative molecular design reshapes drug discovery. Cell Rep Med. 2022. 10.1016/j.xcrm.2022.100794. 36306797 10.1016/j.xcrm.2022.100794 PMC9797947

[24] Olusegun, J., et al., Integration of AI with electronic health records: enhancing clinical workflows. 2024.

[25] Mohsen F, et al. Artificial intelligence-based methods for fusion of electronic health records and imaging data. Sci Rep. 2022;12(1):17981. 36289266 10.1038/s41598-022-22514-4 PMC9605975

[26] Moafa KMY, et al. Artificial intelligence for improved health management: application, uses, opportunities, and challenges-a systematic review. Egypt J Chem. 2024;67(13):865–80.

[27] Rajkomar A, et al. Scalable and accurate deep learning with electronic health records. NPJ Digit Med. 2018;1(1):18. 31304302 10.1038/s41746-018-0029-1 PMC6550175

[28] Friedman C, Elhadad N. Natural language processing in health care and biomedicine. In: Biomedical informatics: computer applications in health care and biomedicine. London: Springer; 2014.

[29] Obermeyer Z, et al. Dissecting racial bias in an algorithm used to manage the health of populations. Science. 2019;366(6464):447–53. 31649194 10.1126/science.aax2342

[30] Davenport T, Kalakota R. The potential for artificial intelligence in healthcare. Future Healthc J. 2019;6(2):94–8. 31363513 10.7861/futurehosp.6-2-94 PMC6616181

[31] He J, et al. The practical implementation of artificial intelligence technologies in medicine. Nat Med. 2019;25(1):30–6. 30617336 10.1038/s41591-018-0307-0 PMC6995276

[32] Mulukuntla S, Venkata SP. AI-driven personalized medicine: assessing the impact of federal policies on advancing patient-centric care. EPH-Int J Med Health Sci. 2020;6(2):20–6.

[33] Parekh A-DE, et al. Artificial intelligence (AI) in personalized medicine: AI-generated personalized therapy regimens based on genetic and medical history. Ann Med Surg. 2023;85(11):5831–3. 10.1097/MS9.0000000000001320 PMC10617817 37915639

[34] Bhattamisra SK, et al. Artificial intelligence in pharmaceutical and healthcare research. Big Data Cogn Comput. 2023;7(1):10.

[35] Quazi S. Artificial intelligence and machine learning in precision and genomic medicine. Med Oncol. 2022;39(8):120. 35704152 10.1007/s12032-022-01711-1 PMC9198206

[36] Vettoretti M, et al. Advanced diabetes management using artificial intelligence and continuous glucose monitoring sensors. Sensors. 2020;20(14):3870. 32664432 10.3390/s20143870 PMC7412387

[37] Jena B, et al. Brain tumor characterization using radiogenomics in artificial intelligence framework. Cancers (Basel). 2022;14(16):4052. 36011048 10.3390/cancers14164052 PMC9406706

[38] Alam MA, et al. The role of predictive analytics in early disease detection: a data-driven approach to preventive healthcare. Innov Eng J. 2024;1(01):105–23.

[39] Alessa A, Faezipour M. A review of influenza detection and prediction through social networking sites. Theor Biol Med Model. 2018;15:1–27. 29386017 10.1186/s12976-017-0074-5 PMC5793414

[40] Srinivasan SM, Sharma V. Applications of AI in cardiovascular disease detection—A review of the specific ways in which AI is being used to detect and diagnose cardiovascular diseases. AI Dis Detect. 2025;1:123–46.

[41] Khalifa M, Albadawy M. AI in diagnostic imaging: revolutionising accuracy and efficiency. Comput Methods Progr Biomed. 2024;5:100146.

[42] Li X, et al. Role of artificial intelligence in medical image analysis: a review of current trends and future directions. J Med Biol Eng. 2024;44(2):231–43.

[43] Megat Ramli PN, et al. A systematic review: the role of artificial intelligence in lung cancer screening in detecting lung nodules on chest X-rays. Diagnostics. 2025;15(3):246. 39941176 10.3390/diagnostics15030246 PMC11817343

[44] Litjens G, et al. A survey on deep learning in medical image analysis. Med Image Anal. 2017;42:60–88. 28778026 10.1016/j.media.2017.07.005

[45] Kumar RR, Priyadarshi R. Denoising and segmentation in medical image analysis: a comprehensive review on machine learning and deep learning approaches. Multimedia Tools Appl. 2025;84(12):10817–75.

[46] Zhu B, et al. Image reconstruction by domain-transform manifold learning. Nature. 2018;555(7697):487–92. 29565357 10.1038/nature25988

[47] Xu H, et al. A whole-slide foundation model for digital pathology from real-world data. Nature. 2024;630(8015):181–8. 38778098 10.1038/s41586-024-07441-w PMC11153137

[48] Schutte K, et al. An artificial intelligence model predicts the survival of solid tumour patients from imaging and clinical data. Eur J Cancer. 2022;174:90–8. 35985252 10.1016/j.ejca.2022.06.055

[49] Wang X, et al. A pathology foundation model for cancer diagnosis and prognosis prediction. Nature. 2024;634(8035):970–8. 39232164 10.1038/s41586-024-07894-z PMC12186853

[50] McKinney SM, et al. International evaluation of an AI system for breast cancer screening. Nature. 2020;577(7788):89–94. 31894144 10.1038/s41586-019-1799-6

[51] Gupta U, et al. The contribution of artificial intelligence to drug discovery: Current progress and prospects for the future. Microbial Data Intell Comput Techn Sustain Comput. 2024;1:1–23.

[52] Fahim YA, Hasani IW, Ragab WM. Promising biomedical applications using superparamagnetic nanoparticles. Eur J Med Res. 2025;30(1):441. 40452035 10.1186/s40001-025-02696-z PMC12128481

[53] Murugan NA, et al. Artificial intelligence in virtual screening: models versus experiments. Drug Discov Today. 2022;27(7):1913–23. 35597513 10.1016/j.drudis.2022.05.013

[54] Wong F, et al. Discovery of a structural class of antibiotics with explainable deep learning. Nature. 2024;626(7997):177–85. 38123686 10.1038/s41586-023-06887-8 PMC10866013

[55] Lopez JS, Banerji U. Combine and conquer: challenges for targeted therapy combinations in early phase trials. Nat Rev Clin Oncol. 2017;14(1):57–66. 27377132 10.1038/nrclinonc.2016.96 PMC6135233

[56] Smith GF. Artificial intelligence in drug safety and metabolism. In: Artificial Intelligence in Drug Design. Springer; 2021. p. 483–501. 10.1007/978-1-0716-1787-8_22 34731484

[57] Zhu H. Big data and artificial intelligence modeling for drug discovery. Annu Rev Pharmacol Toxicol. 2020;60(1):573–89. 31518513 10.1146/annurev-pharmtox-010919-023324 PMC7010403

[58] Ciallella HL, Zhu H. Advancing computational toxicology in the big data era by artificial intelligence: data-driven and mechanism-driven modeling for chemical toxicity. Chem Res Toxicol. 2019;32(4):536–47. 30907586 10.1021/acs.chemrestox.8b00393 PMC6688471

[59] Chan HCS, et al. Advancing drug discovery via artificial intelligence. Trends Pharmacol Sci. 2019;40(8):592–604. 31320117 10.1016/j.tips.2019.06.004

[60] Petitjean, M. and A.C. Camproux, In Silico Medicinal Chemistry: Computational Methods to Support Drug Design. Edited by Nathan Brown. Wiley Online Library. 2016

[61] Pereira JC, Caffarena ER, Dos Santos CN. Boosting docking-based virtual screening with deep learning. J Chem Inf Model. 2016;56(12):2495–506. 28024405 10.1021/acs.jcim.6b00355

[62] Aftab Tariq, Y.H., Advancing Healthcare: The Power of AI in Robotics, Diagnostics, and Precision Medicine.

[63] Abbasi N, Hussain HK. Integration of artificial intelligence and smart technology: AI-driven robotics in surgery: precision and efficiency. J Artif Intell General Sci. 2024;5(1):381–90.

[64] Pinto-Coelho L. How artificial intelligence is shaping medical imaging technology: a survey of innovations and applications. Bioengineering. 2023;10(12):1435. 38136026 10.3390/bioengineering10121435 PMC10740686

[65] Wah JNK. Revolutionizing surgery: AI and robotics for precision, risk reduction, and innovation. J Robot Surg. 2025;19(1):1–15. 10.1007/s11701-024-02205-0 39776281

[66] Khajeh E, et al. Outcomes of robot-assisted surgery in rectal cancer compared with open and laparoscopic surgery. Cancers. 2023;15(3):839. 36765797 10.3390/cancers15030839 PMC9913667

[67] Hopkins MB, et al. Comparing pathologic outcomes for robotic versus laparoscopic surgery in rectal cancer resection: a propensity adjusted analysis of 7616 patients. Surg Endosc. 2020;34(6):2613–22. 31346754 10.1007/s00464-019-07032-1 PMC8117669

[68] Kleinsmit, G.H., Assessment of the impact of new medical technology on teamwork and patient safety in the operating room. 2011.

[69] Kamarajah SK, et al. Robotic versus conventional laparoscopic pancreaticoduodenectomy a systematic review and meta-analysis. Eur J Surg Oncol. 2020;46(1):6–14. 31409513 10.1016/j.ejso.2019.08.007

[70] Meziane F, et al. Intelligent systems in manufacturing: current developments and future prospects. Integr Manuf Syst. 2000;11(4):218–38.

[71] Fahim YA, et al. Biomedical and environmental applications via nanobiocatalysts and enzyme immobilization. Eur J Med Res. 2025;30(1):505. 40544317 10.1186/s40001-025-02782-2 PMC12181844

[72] Kabra A, et al. Computational fluid dynamics used by mixing vessels for predicting hydrodynamic behaviour of mixture: an overview. Mater Today Proc. 2021;47:2305–9.

[73] Rantanen J, Khinast J. The future of pharmaceutical manufacturing sciences. J Pharm Sci. 2015;104(11):3612–38. 26280993 10.1002/jps.24594 PMC4973848

[74] Steiner S, et al. Organic synthesis in a modular robotic system driven by a chemical programming language. Science. 2019;363(6423):eaav2211. 30498165 10.1126/science.aav2211

[75] Faure A, York P, Rowe RC. Process control and scale-up of pharmaceutical wet granulation processes: a review. Eur J Pharm Biopharm. 2001;52(3):269–77. 11677069 10.1016/s0939-6411(01)00184-9

[76] Landin M. Artificial intelligence tools for scaling up of high shear wet granulation process. J Pharm Sci. 2017;106(1):273–7. 27816264 10.1016/j.xphs.2016.09.022

[77] Mak K-K, Pichika MR. Artificial intelligence in drug development: present status and future prospects. Drug Discov Today. 2019;24(3):773–80. 30472429 10.1016/j.drudis.2018.11.014

[78] Paul D, et al. Artificial intelligence in drug discovery and development. Drug Discov Today. 2021;26(1):80–93. 33099022 10.1016/j.drudis.2020.10.010 PMC7577280

[79] Luo M, et al. Micro-/nanorobots at work in active drug delivery. Adv Funct Mater. 2018;28(25):1706100.

[80] Das T, Sultana S. Multifaceted applications of micro/nanorobots in pharmaceutical drug delivery systems: a comprehensive review. Future J Pharm Sci. 2024;10(1):2.

[81] Kazi RNA, et al. Nanomedicine: the effective role of nanomaterials in healthcare from diagnosis to therapy. Pharmaceutics. 2025;17(8):987. 40871010 10.3390/pharmaceutics17080987 PMC12389108

[82] Fu J, Yan H. Controlled drug release by a nanorobot. Nat Biotechnol. 2012;30(5):407–8. 22565965 10.1038/nbt.2206

[83] Sutradhar KB, Sumi CD. Implantable microchip: the futuristic controlled drug delivery system. Drug Deliv. 2016;23(1):1–11. 24758139 10.3109/10717544.2014.903579

[84] Rasa AR. Artificial intelligence and its revolutionary role in physical and mental rehabilitation: a review of recent advancements. BioMed Res Int. 2024;2024(1):9554590. 39720127 10.1155/bmri/9554590 PMC11668540

[85] Nizamis K, et al. Converging robotic technologies in targeted neural rehabilitation: a review of emerging solutions and challenges. Sensors. 2021;21(6):2084. 33809721 10.3390/s21062084 PMC8002299

[86] Anderson D. Artificial intelligence and applications in PM&R. Am J Phys Med Rehabil. 2019;98(11):e128–9. 30839314 10.1097/PHM.0000000000001171

[87] Khalid UB, et al. Impact of AI-powered solutions in rehabilitation process: recent improvements and future trends. Int J Gen Med. 2024. 10.2147/IJGM.S453903. 38495919 10.2147/IJGM.S453903 PMC10944308

[88] Aggarwal, R. and S.S. Ganvir, Artificial intelligence in physiotherapy. Medknow. 2021; 55–57.

[89] Jansen O, et al. Hybrid assistive limb exoskeleton HAL in the rehabilitation of chronic spinal cord injury: proof of concept; the results in 21 patients. World Neurosurg. 2018;110:e73–8. 29081392 10.1016/j.wneu.2017.10.080

[90] Kawamoto H, Sankai Y. Power assist method based on phase sequence and muscle force condition for HAL. Adv Robotics. 2005;19(7):717–34.

[91] Louie DR, Eng JJ. Powered robotic exoskeletons in post-stroke rehabilitation of gait: a scoping review. J Neuroeng Rehabil. 2016;13:1–10. 27278136 10.1186/s12984-016-0162-5 PMC4898381

[92] Al Kuwaiti A, et al. A review of the role of artificial intelligence in healthcare. J Pers Med. 2023;13(6):951. 37373940 10.3390/jpm13060951 PMC10301994

[93] Qiu H, et al. Applications of artificial intelligence in screening, diagnosis, treatment, and prognosis of colorectal cancer. Curr Oncol. 2022;29(3):1773–95. 35323346 10.3390/curroncol29030146 PMC8947571

[94] Sovich JL, Sartor Z, Misra S. Developments in screening tests and strategies for colorectal cancer. BioMed Res Int. 2015;2015(1):326728. 26504799 10.1155/2015/326728 PMC4609363

[95] Rompianesi G, et al. Artificial intelligence in the diagnosis and management of colorectal cancer liver metastases. World J Gastroenterol. 2022;28(1):108. 35125822 10.3748/wjg.v28.i1.108 PMC8793013

[96] Davri A, et al. Deep learning on histopathological images for colorectal cancer diagnosis: a systematic review. Diagnostics. 2022;12(4):837. 35453885 10.3390/diagnostics12040837 PMC9028395

[97] Deepali N, Goel, Khandnor P. Advances in AI-based genomic data analysis for cancer survival prediction. Multim Tools Appl. 2024;1:1–28.

[98] Yamada M, et al. Development of a real-time endoscopic image diagnosis support system using deep learning technology in colonoscopy. Sci Rep. 2019;9(1):14465. 31594962 10.1038/s41598-019-50567-5 PMC6783454

[99] Wan N, et al. Machine learning enables detection of early-stage colorectal cancer by whole-genome sequencing of plasma cell-free DNA. BMC Cancer. 2019;19(1):832. 31443703 10.1186/s12885-019-6003-8 PMC6708173

[100] Rathore S, et al. Novel structural descriptors for automated colon cancer detection and grading. Comput Methods Programs Biomed. 2015;121(2):92–108. 26094859 10.1016/j.cmpb.2015.05.008

[101] Giaquinto AN, et al. Breast cancer statistics, 2022. CA Cancer J Clin. 2022;72(6):524–41. 36190501 10.3322/caac.21754

[102] Filho OM, et al. Impact of HER2 heterogeneity on treatment response of early-stage HER2-positive breast cancer: phase II neoadjuvant clinical trial of T-DM1 combined with pertuzumab. Cancer Discov. 2021;11(10):2474–87. 33941592 10.1158/2159-8290.CD-20-1557 PMC8598376

[103] Dowling GP, et al. Predictive value of pretreatment circulating inflammatory response markers in the neoadjuvant treatment of breast cancer: meta-analysis. Br J Surg. 2024;111(5):znae132. 38801441 10.1093/bjs/znae132 PMC11129713

[104] Ji J, et al. Artificial intelligence-based pathology to assist prediction of neoadjuvant therapy responses for breast cancer. Cancer Med. 2025;14(15):e71132. 40762329 10.1002/cam4.71132 PMC12322828

[105] Cruz-Roa A, et al. Accurate and reproducible invasive breast cancer detection in whole-slide images: a deep learning approach for quantifying tumor extent. Sci Rep. 2017;7(1):46450. 28418027 10.1038/srep46450 PMC5394452

[106] Han Z, et al. Breast cancer multi-classification from histopathological images with structured deep learning model. Sci Rep. 2017;7(1):4172. 28646155 10.1038/s41598-017-04075-z PMC5482871

[107] Luo J, et al. A deep-learning-based clinical risk stratification for overall survival in adolescent and young adult women with breast cancer. J Cancer Res Clin Oncol. 2023;149(12):10423–33. 37277578 10.1007/s00432-023-04955-0 PMC11798295

[108] Huang CY, et al. Deep-learning model to improve histological grading and predict upstaging of atypical ductal hyperplasia/ductal carcinoma in situ on breast biopsy. Histopathology. 2024;84(6):983–1002. 38288642 10.1111/his.15144

[109] Siegel RL, et al. Cancer statistics, 2022. CA Cancer J Clin. 2022. 10.3322/caac.21708.36346402

[110] Yan W, et al. Applicability analysis of immunotherapy for lung cancer patients based on deep learning. Methods. 2022;205:149–56. 35809770 10.1016/j.ymeth.2022.07.004

[111] Weber D, et al. Accurate detection of tumor-specific gene fusions reveals strongly immunogenic personal neo-antigens. Nat Biotechnol. 2022;40(8):1276–84. 35379963 10.1038/s41587-022-01247-9 PMC7613288

[112] Gao Q, et al. The artificial intelligence and machine learning in lung cancer immunotherapy. J Hematol Oncol. 2023;16(1):55. 37226190 10.1186/s13045-023-01456-y PMC10207827

[113] Ashraf SF, et al. Predicting benign, preinvasive, and invasive lung nodules on computed tomography scans using machine learning. J Thorac Cardiovasc Surg. 2022;163(4):1496–505. 33726909 10.1016/j.jtcvs.2021.02.010

[114] Wang S, et al. Artificial intelligence in lung cancer pathology image analysis. Cancers. 2019;11(11):1673. 31661863 10.3390/cancers11111673 PMC6895901

[115] Zhong Y, et al. Deep learning for prediction of N2 metastasis and survival for clinical stage I non–small cell lung cancer. Radiology. 2022;302(1):200–11. 34698568 10.1148/radiol.2021210902

[116] Prelaj A, et al. Machine learning using real-world and translational data to improve treatment selection for NSCLC patients treated with immunotherapy. Cancers (Basel). 2022;14(2):435. 35053597 10.3390/cancers14020435 PMC8773718

[117] Bacha A, Shah HH. Ai-powered virtual health assistants: transforming patient care and engagement. Glob Insights Artif Intell Comput. 2025;1(1):15–30.

[118] Jungmann SM, et al. Accuracy of a chatbot (Ada) in the diagnosis of mental disorders: comparative case study with lay and expert users. JMIR Form Res. 2019;3(4):e13863. 31663858 10.2196/13863 PMC6914276

[119] Bickmore TW, et al. ‘It’s just like you talk to a friend’relational agents for older adults. Interact Comput. 2005;17(6):711–35.

[120] De Choudhury, M., et al. Predicting depression via social media.

[121] Miner AS, et al. Smartphone-based conversational agents and responses to questions about mental health, interpersonal violence, and physical health. JAMA Intern Med. 2016;176(5):619–25. 26974260 10.1001/jamainternmed.2016.0400 PMC4996669

[122] Secara I-A, Hordiiuk D. Personalized health monitoring systems: integrating wearable and AI. J Intell Learn Syst Appl. 2024;16(2):44–52.

[123] Kang HS, Exworthy M. Wearing the future—wearables to empower users to take greater responsibility for their health and care: scoping review. JMIR Mhealth Uhealth. 2022;10(7):e35684. 35830222 10.2196/35684 PMC9330198

[124] Lu L, et al. Wearable health devices in health care: narrative systematic review. JMIR Mhealth Uhealth. 2020;8(11):e18907. 33164904 10.2196/18907 PMC7683248

[125] George ASH, Shahul A, George AS. Wearable sensors: A new way to track health and wellness. Partn Universal Int Innov J. 2023;1(4):15–34.

[126] Salinari A, et al. The application of digital technologies and artificial intelligence in healthcare: an overview on nutrition assessment. Dis. 2023;11(3):97. 10.3390/diseases11030097 PMC10366918 37489449

[127] Sorrentino FS, et al. Novel approaches for early detection of retinal diseases using artificial intelligence. J Pers Med. 2024;14(7):690. 39063944 10.3390/jpm14070690 PMC11278069

[128] Liu Y, et al. Artificial intelligence-based breast cancer nodal metastasis detection: insights into the black box for pathologists. Arch Pathol Lab Med. 2019;143(7):859–68. 30295070 10.5858/arpa.2018-0147-OA

[129] Qureshi A. Robotic surgery: enhancing precision in complex procedures. Rev J Neurol Med Sci Rev. 2024;2(3):28–36.

[130] Ismail AR, et al. Utilising VGG-16 of convolutional neural network for medical image classification. Int J Percept Cognit Comput. 2024;10(1):113–8.

[131] Cull J, et al. Epic sepsis model inpatient predictive analytic tool: a validation study. Crit Care Explor. 2023;5(7):e0941. 37405252 10.1097/CCE.0000000000000941 PMC10317482

[132] dos Santos FM, et al. Comparing artificial intelligence AI-rad companion chest CT vs experts on emphysema, lung nodules, thoracic aorta and thoracic spine. Acta Radiológica Portuguesa. 2024;36(1):6–11.

[133] Desai D, et al. Review of AlphaFold 3: transformative advances in drug design and therapeutics. Cureus. 2024;16:7. 10.7759/cureus.63646 PMC11292590 39092344

[134] Yang H-C, et al. Deep learning for automated segmentation of brain edema in meningioma after radiosurgery. BMC Med Imaging. 2025;25(1):130. 40264119 10.1186/s12880-025-01660-x PMC12016358

[135] Liu C, et al. Using artificial intelligence (Watson for Oncology) for treatment recommendations amongst Chinese patients with lung cancer: feasibility study. J Med Internet Res. 2018;20(9):e11087. 30257820 10.2196/11087 PMC6231834

[136] Zhang Y, et al. Lung nodule detectability of artificial intelligence-assisted CT image reading in lung cancer screening. Curr Med Imaging Rev. 2022;18(3):327–34. 10.2174/1573405617666210806125953 34365951

[137] Tayefi M, et al. Challenges and opportunities beyond structured data in analysis of electronic health records. Wiley Interdiscip Rev Comput Stat. 2021;13(6):e1549.

[138] Ortega-Calvo AS, et al. Aimdp: an artificial intelligence modern data platform. Use case for Spanish national health service data silo. Future Gener Comput Syst. 2023;143:248–64.

[139] Williams E, et al. A standardized clinical data harmonization pipeline for scalable AI application deployment (FHIR-DHP): validation and usability study. JMIR Med Inform. 2023;11:e43847. 36943344 10.2196/43847 PMC10131740

[140] Sinaci AA, et al. A data transformation methodology to create findable, accessible, interoperable, and reusable health data: software design, development, and evaluation study. J Med Internet Res. 2023;25:e42822. 36884270 10.2196/42822 PMC10034606

[141] Cai Q, et al. A survey on multimodal data-driven smart healthcare systems: approaches and applications. IEEE Access. 2019;7:133583–99.

[142] Althati C, Tomar M, Shanmugam L. Enhancing data integration and management: the role of AI and machine learning in modern data platforms. J Artif Intell General Sci. 2024;2(1):220–32.

[143] Pashazadeh A, Navimipour NJ. Big data handling mechanisms in the healthcare applications: a comprehensive and systematic literature review. J Biomed Inform. 2018;82:47–62. 29655946 10.1016/j.jbi.2018.03.014

[144] Kalia K, Gupta N. Analysis of hadoop mapreduce scheduling in heterogeneous environment. Ain Shams Eng J. 2021;12(1):1101–10.

[145] Khalil WA, Torkey H, Attiya G. Survey of Apache Spark optimized job scheduling in Big Data. Int J Ind Sustain Dev. 2020;1(1):39–48.

[146] Surianarayanan C, et al. A survey on optimization techniques for edge artificial intelligence (AI). Sensors. 2023;23(3):1279. 36772319 10.3390/s23031279 PMC9919555

[147] Hua H, et al. Edge computing with artificial intelligence: a machine learning perspective. ACM Comput Surv. 2023;55(9):1–35.

[148] Khanh QV, et al. An integrating computing framework based on edge-fog-cloud for internet of healthcare things applications. Internet Things. 2023;23:100907.

[149] Wani NA, Kumar R, Bedi J. Deepxplainer: an interpretable deep learning based approach for lung cancer detection using explainable artificial intelligence. Comput Methods Programs Biomed. 2024;243:107879. 37897989 10.1016/j.cmpb.2023.107879

[150] Kök I, et al. Explainable artificial intelligence (XAI) for internet of things: a survey. IEEE Internet Things J. 2023;10(16):14764–79.

[151] Band SS, et al. Application of explainable artificial intelligence in medical health: a systematic review of interpretability methods. Inf Med Unlocked. 2023;40:101286.

[152] Liu B. Lifelong machine learning: a paradigm for continuous learning. Front Comput Sci. 2017;11(3):359–61.

[153] Feng J, et al. Clinical artificial intelligence quality improvement: towards continual monitoring and updating of AI algorithms in healthcare. NPJ Digit Med. 2022;5(1):66. 35641814 10.1038/s41746-022-00611-y PMC9156743

[154] Garg SK, et al. Accuracy and safety of Dexcom G7 continuous glucose monitoring in adults with diabetes. Diabetes Technol Ther. 2022;24(6):373–80. 35157505 10.1089/dia.2022.0011 PMC9208857

[155] Allam, Z., The rise of machine intelligence in the COVID-19 pandemic and its impact on health policy. Surveying the COVID-19 Pandemic and its Implications, 2020: 89.

[156] Prabhu, A., et al., Online continual learning without the storage constraint. arXiv preprint arXiv:2305.09253, 2023.

[157] Hu Y, et al. Artificial intelligence security: threats and countermeasures. ACM Comput Surv. 2021;55(1):1–36.

[158] Qayyum A, et al. Secure and robust machine learning for healthcare: a survey. IEEE Rev Biomed Eng. 2020;14:156–80. 10.1109/RBME.2020.3013489 32746371

[159] Goktas P, Grzybowski A. Shaping the future of healthcare: ethical clinical challenges and pathways to trustworthy AI. J Clin Med. 2025;14(5):1605. 40095575 10.3390/jcm14051605 PMC11900311

[160] Ueda D, et al. Fairness of artificial intelligence in healthcare: review and recommendations. Jpn J Radiol. 2024;42(1):3–15. 37540463 10.1007/s11604-023-01474-3 PMC10764412

[161] Pagano TP, et al. Bias and unfairness in machine learning models: a systematic review on datasets, tools, fairness metrics, and identification and mitigation methods. Big Data Cogn Comput. 2023;7(1):15.

[162] Kucukkaya A, et al. Equality, diversity, and inclusion in artificial intelligence-driven healthcare chatbots addressing challenges and shaping strategies. Eur J Cardiovasc Nurs. 2025;1:105. 10.1093/eurjcn/zvaf104 40455916

[163] Gao X, et al. Artificial intelligence applications in smart healthcare: a survey. Futur Int. 2024;16(9):308.

[164] Wittig, A. and M. Wittig, *Amazon Web Services in Action: An in-depth guide to AWS*. 2023: Simon and Schuster.

[165] Liang, M., et al. Mystique: Enabling accurate and scalable generation of production ai benchmarks.

[166] Amjad A, Kordel P, Fernandes G. A review on innovation in healthcare sector (telehealth) through artificial intelligence. Sustainability. 2023;15(8):6655.

[167] Uschnig C, et al. Tele-ultrasound in the era of COVID-19: a practical guide. Ultrasound Med Biol. 2022;48(6):965–74. 35317949 10.1016/j.ultrasmedbio.2022.01.001 PMC8743597

[168] Rao DP, et al. Evaluation of an offline, artificial intelligence system for referable glaucoma screening using a smartphone-based fundus camera: a prospective study. Eye. 2024;38(6):1104–11. 38092938 10.1038/s41433-023-02826-z PMC11009383

[169] Wang C-H, et al. Lightweight deep learning: an overview. IEEE Consum Electron Mag. 2022;13(4):51–64.

[170] Henrich J, Heine SJ, Norenzayan A. The weirdest people in the world? Behav Brain Sci. 2010;33(2–3):61–83. 20550733 10.1017/S0140525X0999152X

[171] Ellwood-Lowe ME, et al. Time-varying effects of income on hippocampal volume trajectories in adolescent girls. Dev Cogn Neurosci. 2018;30:41–50. 29275097 10.1016/j.dcn.2017.12.005 PMC5963716

[172] Hackman DA, Farah MJ, Meaney MJ. Socioeconomic status and the brain: mechanistic insights from human and animal research. Nat Rev Neurosci. 2010;11(9):651–9. 20725096 10.1038/nrn2897 PMC2950073

[173] Neto, E.C., Using permutations to detect, quantify and correct for confounding in machine learning predictions. arXiv preprint arXiv:1805.07465, 2018.

[174] He H, Garcia EA. Learning from imbalanced data. IEEE Trans Knowl Data Eng. 2009;21(9):1263–84.

[175] Alsheibani, S.A., D. Cheung, and D. Messom, Factors inhibiting the adoption of artificial intelligence at organizational level: A preliminary investigation. Twenty-fifth Americas Conference on Information Systems, Cancun. 2019.

[176] Lee, M.K. and K. Rich. Who is included in human perceptions of AI?: Trust and perceived fairness around healthcare AI and cultural mistrust.

[177] Strohm L, et al. Implementation of artificial intelligence (AI) applications in radiology: hindering and facilitating factors. Eur Radiol. 2020;30(10):5525–32. 32458173 10.1007/s00330-020-06946-y PMC7476917

[178] Cadario R, Longoni C, Morewedge CK. Understanding, explaining, and utilizing medical artificial intelligence. Nat Hum Behav. 2021;5(12):1636–42. 34183800 10.1038/s41562-021-01146-0

[179] Unanah OV, Aidoo EM. The potential of AI technologies to address and reduce disparities within the healthcare system by enabling more personalized and efficient patient engagement and care management. World J Adv Res Rev. 2025;25(2):2643–64.

[180] Amann J, et al. Explainability for artificial intelligence in healthcare: a multidisciplinary perspective. BMC Med Inform Decis Mak. 2020;20(1):310. 33256715 10.1186/s12911-020-01332-6 PMC7706019

[181] Chen RJ, et al. Algorithmic fairness in artificial intelligence for medicine and healthcare. Nat Biomed Eng. 2023;7(6):719–42. 37380750 10.1038/s41551-023-01056-8 PMC10632090

[182] Cross JL, Choma MA, Onofrey JA. Bias in medical AI: implications for clinical decision-making. PLoS Digit Health. 2024;3(11):e0000651. 39509461 10.1371/journal.pdig.0000651 PMC11542778

[183] Jurczuk M, Suprunowicz M. Consent in data privacy: a general comparison of GDPR and HIPAA. Przegląd Prawniczy Uniwersytetu im Adam Mickiewicza. 2024;16:173–94.

[184] Addario BJ, et al. Patient value: perspectives from the advocacy community. Health Expect. 2018;21(1):57–63. 28940536 10.1111/hex.12628 PMC5750698

[185] Ali H. Artificial intelligence in multi-omics data integration: Advancing precision medicine, biomarker discovery and genomic-driven disease interventions. Int J Sci Res Arch. 2023;8(1):1012–30.

[186] Ali H. Quantum computing and AI in healthcare: Accelerating complex biological simulations, genomic data processing, and drug discovery innovations. World J Adv Res Rev. 2023;20(2):1466–84.

[187] Reddy S. Global harmonization of artificial intelligence-enabled software as a medical device regulation: addressing challenges and unifying standards. Mayo Clinic Proc Digit Health. 2025. 10.1016/j.mcpdig.2024.100191. 10.1016/j.mcpdig.2024.100191 PMC11975980 40207007

[188] Palaniappan K, Lin EYT, Vogel S. Global regulatory frameworks for the use of artificial intelligence (AI) in the healthcare services sector. Healthcare. 2024. 10.3390/healthcare12050562. 38470673 10.3390/healthcare12050562 PMC10930608

[189] Ngcobo M. The ethics and law of medical AI in South Africa: balancing innovation with responsibility. SAMJ S Afr Med J. 2025;115(5B):75–9. 10.7196/SAMJ.2025.v115i5b.3667 41378646

[190] Signé, L., Strategies for effective health care for Africa in the Fourth Industrial Revolution. Brookings, 2021.

[191] Rehm HL, et al. GA4GH: international policies and standards for data sharing across genomic research and healthcare. Cell Genom. 2021. 10.1016/j.xgen.2021.100029. 35072136 10.1016/j.xgen.2021.100029 PMC8774288

